# Diabetes causes marked inhibition of mitochondrial metabolism in pancreatic β-cells

**DOI:** 10.1038/s41467-019-10189-x

**Published:** 2019-06-06

**Authors:** Elizabeth Haythorne, Maria Rohm, Martijn van de Bunt, Melissa F. Brereton, Andrei I. Tarasov, Thomas S. Blacker, Gregor Sachse, Mariana Silva dos Santos, Raul Terron Exposito, Simon Davis, Otto Baba, Roman Fischer, Michael R. Duchen, Patrik Rorsman, James I. MacRae, Frances M. Ashcroft

**Affiliations:** 1 Department of Physiology, Anatomy and Genetics and OXION, University of Oxford, Oxford, OX1 3PT UK; 2 Oxford Centre for Diabetes, Endocrinology and Metabolism, University of Oxford, Churchill Hospital, Oxford, OX3 7EL UK; 3 Department of Cell and Developmental Biology, University College London, Gower Street, London, WC1E 6BT UK; 4 The Francis Crick Institute, 1 Midland Road, London, NW1 1AT UK; 5 Discovery Proteomics Facility, Target Discovery Institute, University of Oxford, Oxford, OX3 7FZ UK; 6 Tokushima University Graduate School, 3-18-15, Kuramoto-cho, Tokushima, 770-8504 Japan; 7 Metabolic Research, Department of Neuroscience and Physiology, Sahlgrenska Academy, University of Göteborg, Box 433, 40530 Göteborg, Sweden; 8 Institute for Diabetes and Cancer (IDC), Helmholtz Center Munich, Neuherberg, 85764 Germany; 9 Present Address: Department of Bioinformatics and Data Mining, Novo Nordisk A/S, Måløv, 2760 Denmark

**Keywords:** Metabolism, Diabetes

## Abstract

Diabetes is a global health problem caused primarily by the inability of pancreatic β-cells to secrete adequate levels of insulin. The molecular mechanisms underlying the progressive failure of β-cells to respond to glucose in type-2 diabetes remain unresolved. Using a combination of transcriptomics and proteomics, we find significant dysregulation of major metabolic pathways in islets of diabetic βV59M mice, a non-obese, eulipidaemic diabetes model. Multiple genes/proteins involved in glycolysis/gluconeogenesis are upregulated, whereas those involved in oxidative phosphorylation are downregulated. In isolated islets, glucose-induced increases in NADH and ATP are impaired and both oxidative and glycolytic glucose metabolism are reduced. INS-1 β-cells cultured chronically at high glucose show similar changes in protein expression and reduced glucose-stimulated oxygen consumption: targeted metabolomics reveals impaired metabolism. These data indicate hyperglycaemia induces metabolic changes in β-cells that markedly reduce mitochondrial metabolism and ATP synthesis. We propose this underlies the progressive failure of β-cells in diabetes.

## Abstract (web summary)

Pancreatic beta-cell glucose metabolism is coupled to insulin secretion. Here the authors set out to characterize changes in beta-cell metabolism in hyperglycemia which may contribute to insufficient insulin secretion in type 2 diabetes and, using a multi-omics approach, find that mitochondrial metabolism is perturbed.

## Introduction

Type 2 diabetes (T2D) is a major global public health problem. A complex multifactorial disease, it involves both genetic and lifestyle factors that lead to a gradual deterioration in insulin secretion from pancreatic β-cells and chronic elevation of plasma glucose levels^1,2^. Chronic hyperglycaemia impairs β-cell function^3–6^ and thus may be expected to contribute to the progressive nature of the disease.

Evidence suggests that the primary cause of the insufficient insulin secretion in T2D is impaired metabolism–secretion coupling rather than β-cell loss. Although β-cell mass diminishes in diabetes, it is not sufficient to account for the extent of reduction in insulin secretion^6–8^. This is also demonstrated by the rapid reversal of T2D following bariatric surgery^9^ or a low-calorie diet^10^, and the ability of neonatal diabetes patients with K_ATP_ channel mutations to transfer to sulfonylurea therapy, even after many years of diabetes^11^.

In non-diabetic β-cells, glucose metabolism couples changes in blood glucose to changes in insulin secretion^2,12–14^. Glucose uptake and its subsequent phosphorylation by glucokinase to glucose 6-phosphate drives β-cell glycolysis and pyruvate generation. Pyruvate enters mitochondria where it is oxidised in the tricarboxylic acid (TCA) cycle to produce reducing equivalents (NADH and FADH_2_) that are utilised by the electron transport chain (ETC) to generate a proton gradient that drives ATP synthesis. Glycolytically derived NADH is also important for ATP synthesis as it can provide reducing equivalents to the ETC via the glycerol-3-phosphate and malate–aspartate shuttles. Simultaneous inhibition of both these shuttles strongly reduces insulin release^15,16^. Mitochondrially generated ATP closes K_ATP_ channels in the β-cell plasma membrane and thereby leads to membrane depolarisation, opening of voltage-gated calcium channels, calcium entry and insulin exocytosis^17^. Additional mitochondrially derived coupling factors, such as NADH, citrate and glutamate, have been implicated in the amplification of insulin secretion^13^. The pivotal role of mitochondrial metabolism is illustrated by the fact that glucose-stimulated insulin secretion (GSIS) is impaired by inhibitors of mitochondrial metabolism, by mitochondrial uncouplers or mitochondrial dysfunction^12,13^.

The question of how β-cell metabolism–secretion coupling is impaired in T2D is still largely unresolved. Multiple contributors have been proposed, including impaired glucose metabolism itself, oxidative stress, endoplasmic reticulum stress, impaired exocytosis and β-cell dedifferentiation^12,18^. While all of these may be contributory factors, a crucial question is which is the initial key event that drives diabetes progression. Accumulating evidence suggests this may be impaired β-cell metabolism, as changes in metabolic genes, or in metabolism, have been identified in islets isolated from T2D donors^19,20^, control human islets cultured at 25 mM glucose^3^, diabetic GK rat islets^21^, mouse models of diabetes^22–24^ and insulin-secreting cell lines exposed to high glucose^4,5^. A gene encoding a protein that controls translation of mitochondrial proteins^25^ has also been linked to T2D. These findings add strength to the idea that impaired β-cell metabolism has a pathogenic role in the development of human T2D.

We have explored this idea in detail using a comprehensive multi-omics approach coupled with functional analysis of mitochondrial metabolism. We find that both diabetes and hyperglycaemia cause striking changes in metabolism in pancreatic β-cells. The increase in mitochondrial metabolism that normally occurs in response to glucose is abrogated, leading to a failure of glucose to elevate NADH or increase the rate of ATP synthesis. This is associated with marked changes in the expression of multiple metabolic genes and proteins, most notably those involved in the TCA cycle and ETC. Our data provide a mechanistic explanation for the impaired metabolism and insulin secretion produced by chronic hyperglycaemia and diabetes. We propose that altered β-cell metabolism may be the crucial event that drives diabetes progression.

## Results

### Animal model

To examine the effects of diabetes on β-cell metabolism, we used the βV59M mouse model in which tamoxifen-inducible expression of a constitutively open ATP-sensitive potassium (K_ATP_) channel specifically in pancreatic β-cells renders the β-cell electrically silent and inhibits insulin secretion^22^. Mutant gene expression was induced at 12 weeks of age and mice developed hyperglycaemia (blood glucose levels >20 mM) within 24 h. Islets were isolated 2 weeks later. After 2 weeks of diabetes, blood glucose remained elevated and plasma lipid levels were not significantly altered^26^: thus the changes we observe are due to hyperglycaemia/hypoinsulinaemia and not a secondary consequence of altered lipid metabolism. The βV59M mouse carries a K_ATP_ channel mutation found in patients with neonatal diabetes^22^ and is a good model both for this disease and for the effects of chronic hyperglycaemia seen in other types of diabetes.

### Transcriptomics and proteomics analyses

We performed transcriptomics and proteomics analysis of islets isolated from control mice and βV59M mice that had been diabetic for 2 weeks.

RNA sequencing identified 13,362 protein-coding or lincRNA transcripts in all samples. Of these, 2795 (~20%) were significantly (false discovery rate (FDR) < 1%) altered in 2-week diabetic islets, when compared to control islets (Supplementary Fig. 1a). Expression of 512 genes increased >2-fold in diabetic islets (>1.5-fold for 943 genes), while that of 362 genes decreased >2-fold (882 genes by >1.5-fold).

To determine the relationship between the islet transcriptome and proteome, we performed global protein profiling using liquid chromatography tandem mass spectrometry (LC-MS/MS). A total of 3066 proteins were identified in both data sets, of which 1281 (42%) were significantly different between control and 2-week diabetic islets (analysis of variance (ANOVA), *p* < 0.05). Despite the >4-fold difference in the numbers of genes/proteins identified, which largely reflects method sensitivity, there was a high correlation between significantly changed genes and proteins (*R*^2^ = 0.59, Pearson correlation: Supplementary Fig. 1b).

Pathway analysis of the transcriptomics data identified 89 gene sets with significantly different expression (FDR < 5%) between control and diabetic islets: of these, 59 were enriched for higher expression in diabetic islets compared to controls and 30 for lower expression (Supplementary Fig. 1c). Pathway analysis of the proteomics data identified 69 gene sets with significantly different expression (52 up in diabetes, and 17 up in control) (Supplementary Fig. 1c).

### Metabolic gene and protein expression

**Fig. 1:**
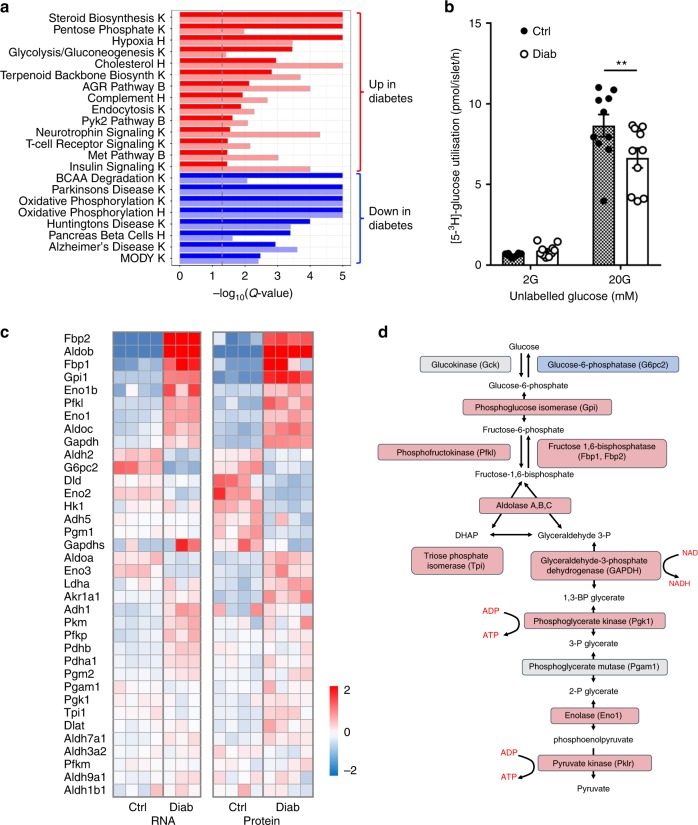
Diabetes alters metabolic pathways. **a** Heat map of pathway enrichment, obtained by simultaneous analysis of transcriptome and proteome data sets using KEGG (K), Hallmark (H) or Biocarta (B). Red, pathways upregulated in diabetic islets. Blue, pathways downregulated in diabetic islets. Dark colours indicate proteomics, pale colours transcriptomics. The dashed vertical line indicates the level of significant change (false discovery rate-adjusted *p* value). PYK2, proline-rich tyrosine kinase 2. MET, tyrosine kinase MET. BCAA, branched chain amino acids. MODY, maturity onset diabetes of the young. **b** Glucose utilisation measured as the production of ^3^H_2_O from [^3^H]-glucose in control islets (hatched, *n* = 10 replicates, islets from 4 mice) and 2-week diabetic βV59M islets (white, *n* = 10, 6 mice) at 2 mM glucose (2 G) and 20 mM glucose (20 G). **p < 0.01 (two-way analysis of variance). Data are mean ± s.e.m. **c** Heat maps of relative mRNA and protein expression of the indicated genes in islets isolated from control (Ctrl) and 2-week diabetic (Diab) βV59M mice. Each box indicates a separate animal. Colour indicates log_2_ fold-change for diabetic vs control islets. **d** Glycolytic pathway. Elevated protein expression levels shown in red, decreased protein levels in blue. Grey, no change or not detected

**Fig. 2:**
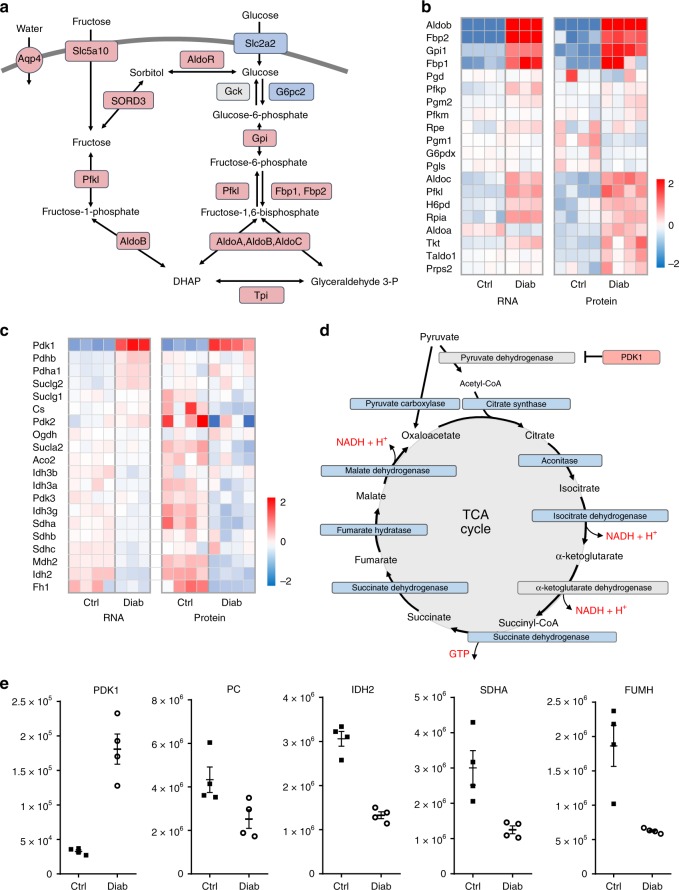
Diabetes upregulates polyol pathway, pentose phosphate pathway and tricarboxylic acid (TCA) cycle genes and proteins. **a** Polyol pathway. Red, proteins upregulated in diabetes. Blue, proteins downregulated in diabetes. Grey, no change or not detected. AldoR, aldolase reductase. Slc2a2, glucose transporter 2. SORD3, sorbitol dehydrogenase. Aqp4, aquaporin 4. **b** Heat maps of mRNA and protein expression of the indicated genes in the pentose phosphate pathway in islets isolated from control (Ctrl) and 2-week diabetic βV59M (Diab) mice. Each box corresponds to a different animal. Colour indicates log_2_ fold-change. **c** Heat maps of relative mRNA and protein levels of the indicated genes in islets isolated from control and 2-week diabetic βV59M mice. Each box corresponds to a different animal. Colour indicates log_2_ fold-change. **d** TCA cycle. Red, proteins upregulated in diabetes; blue, proteins downregulated in diabetes. Grey, no change or not detected. PDK1, pyruvate dehydrogenase kinase 1. **e** Abundance of the indicated proteins, quantified by mass spectrometry, in islets isolated from control (black, Ctrl, *n* = 4) and 2-week diabetic βV59M (white, Diab, *n* = 4) mice. PDK1, pyruvate dehydrogenase kinase 1. PC, pyruvate carboxylase. IDH2, isocitrate dehydrogenase 2. SDHA, succinate dehydrogenase. FUMH, fumarate hydratase (mitochondrial). Each data point indicates a separate mouse. Mean ± s.e.m. is indicated

Figure 1a shows gene sets that were significantly enriched in the same direction at both the mRNA and protein level by diabetes, identified from the combined results of pathway analysis of both transcriptomics and proteomics data sets. There was considerable overlap in gene sets that were significantly enriched in the RNAseq and proteomics data—of the 24 gene sets that were enriched in both data types, 22 (92%) were directionally consistent. Metabolic pathways were particularly strongly affected (Fig. 1a). Almost all glycolytic enzymes were robustly upregulated in diabetic islets (Fig. 1c, d), and the polyol pathway (Fig. 2a), the pentose phosphate pathway (PPP, Fig. 2b) and steroid/cholesterol biosynthesis (Fig. 1a, Supplementary Fig. 2a) were also strongly upregulated. Indeed, aldolase B was the most upregulated of all proteins (65-fold) and there was also a dramatic increase in both mRNA (246-fold) and protein levels (40-fold) of the fructose/glucose transporter SLC5A10 (Fig. 2a, b and Supplementary Table 1).

**Fig. 3:**
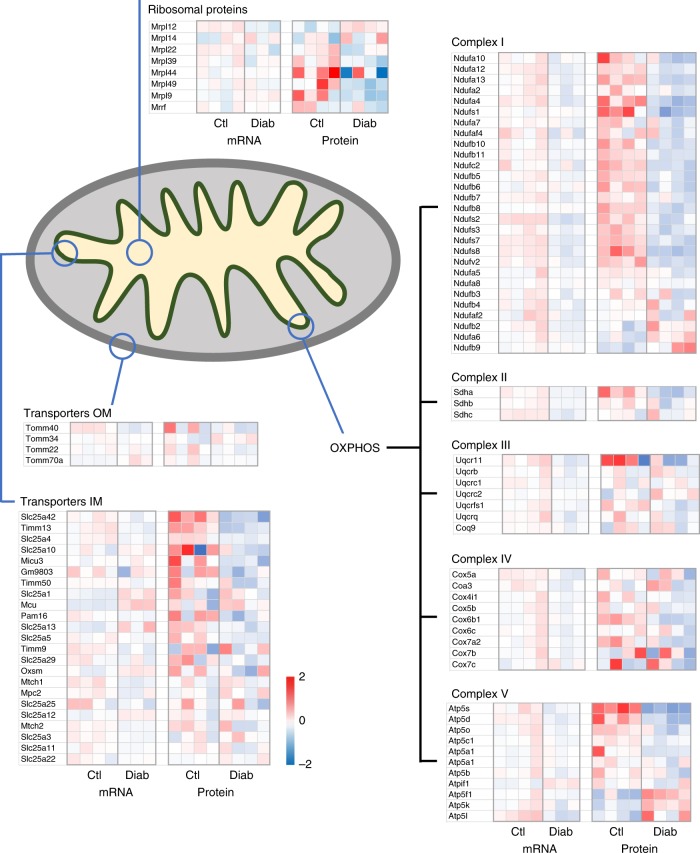
Mitochondrial signatures in control (Ctl) and diabetic (Diab) islets. Heat maps of mRNA and protein levels of the indicated mitochondrial genes in islets isolated from control and 2-week diabetic βV59M mice. Each box represents a different animal. Colour indicates log_2_ fold-change

Conversely, pathways involved in oxidative phosphorylation and branched chain amino acid metabolism were markedly reduced (Fig. 1a). Among the mitochondrial proteins altered in diabetic islets (135/277 identified), 49% were downregulated and 8% upregulated. These included key enzymes involved in the TCA cycle, the ETC, β-oxidation, protein synthesis and transport across the inner mitochondrial membrane (Figs. 2 and 3). Almost all proteins in the TCA cycle were ~2-fold less abundant in diabetic islets (Fig. 2c–e). These included citrate synthase (2.5-fold), succinate dehydrogenase (2.4-fold) and fumarate hydratase (3-fold) (Fig. 2e, Supplementary Table 1). In addition, pyruvate dehydrogenase kinase (PDK1), which inhibits pyruvate dehydrogenase and thereby limits pyruvate entry into the TCA cycle (Fig. 2d), increased 5.5-fold (Fig. 2e, Supplementary Table 1). In β-cells, ~50% of pyruvate enters the TCA cycle via conversion to oxaloacetate by the enzyme pyruvate carboxylase (PC) and this input correlates with glucose-induced insulin secretion^27^. As PC was downregulated 1.7-fold (Fig. 2e, Supplementary Table 1), anaplerotic pyruvate flux is also expected to be reduced in diabetic islets.

Many genes/proteins involved in oxidative phosphorylation were also downregulated (Fig. 3). For example, of the 34 proteins identified in Complex I of the ETC, 17 were significantly downregulated (63%), 3 were unchanged and 2 were upregulated. We also identified 11 proteins that contribute to ATP synthase (Complex V), of which five were significantly downregulated and two upregulated.

While outer mitochondrial membrane transporters were largely unchanged, some inner mitochondrial transport proteins, such as SLC25A42 (a Coenzyme A importer) or TIMM13 (a protein importer), were decreased in diabetic islets (Fig. 3). Two key enzymes of the glycerol phosphate shuttle, which transfers electrons from NADH generated in glycolysis to Complex II of the electron transport chain, (GPD1 and GPD2), were upregulated at both the mRNA and protein level (Supplementary Table 1). In contrast, most enzymes of the malate–aspartate shuttle were unchanged, while ARALAR2 (the mitochondrial aspartate–glutamate transporter) and MDH2 (the mitochondrial malate dehydrogenase) were significantly decreased at the protein level (1.5-fold and 1.9-fold, respectively; Supplementary Table 1).

### Mitochondrial metabolism is impaired in diabetic islets

**Fig. 4:**
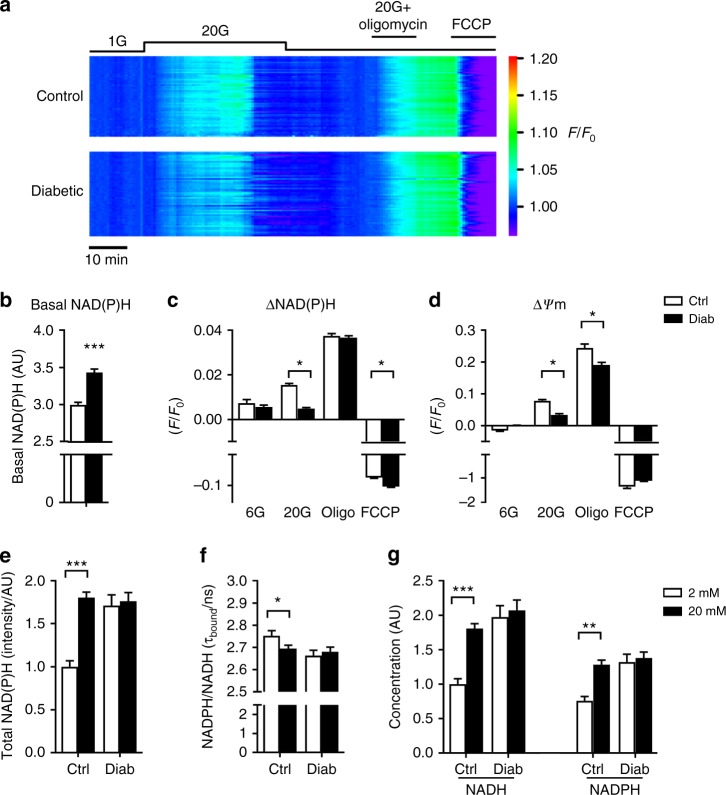
Diabetes alters islet NAD(P)H generation. **a** Relative change in NAD(P)H fluorescence (*F*/*F*_0_) recorded simultaneously in groups of control (above, *n* = 58) or 2-week diabetic βV59M (below, *n* = 54) islets. Each trace represents an individual islet. Data are colour coded according to a ‘rainbow’ LUT, i.e. from violet (low value) to red (high value). **b** Basal NAD(P)H autofluoresence at 2 mM glucose in control (white, *n* = 396 islets) and 2-week diabetic islets (black, *n* = 200 islets). Mean ± s.e.m., *t* test ***p < 0.001. **c** Change in NAD(P)H autofluoresence in response to glucose (6 or 20 mM), to 20 mM glucose + 10 µM oligomycin and to 20 mM glucose + 4 µM FCCP in control (white, *n* = 60–257 islets) and 2-week diabetic βV59M islets (black, *n* = 152–194 islets). **d** Change in mitochondrial membrane potential, as assessed by TMRE (tetramethylrhodamine ethyl ester) fluorescence, in response to glucose (6 or 20 mM), to 20 mM glucose + 10 µM oligomycin and to 20 mM glucose + 4 µM FCCP in control (black, *n* = 83–199 islets) and 2-week diabetic islets (red, *n* = 111–248 islets). **c**, **d** Data are normalised to the level in 2 mM glucose. Mean ± s.e.m. Kruskal–Wallis analysis of variance with Dunn post hoc test. *p < 0.05. **e**–**g** NADH and NADPH autofluorescence measured with fluorescence lifetime imaging microscopy in control (*n* = 21) and 2-week diabetic islets (*n* = 30) exposed to 2 (white bars) or 20 mM (black bars) glucose. Mean ± s.e.m. *t* test *p < 0.05, **p < 0.01, ***p < 0.001. **e** NAD(P)H autofluorescence. **f** Fluorescence lifetime of enzyme-bound NADH + NADPH autofluorescence signal. **g** Relative NADH and NADPH concentrations

Taken together, the changes in mitochondrial gene and protein expression we observe suggest that diabetes/hyperglycaemia causes a marked reduction in oxidative metabolism. To determine whether this is the case, we measured the total autofluorescence from NADH and NADPH (referred to as NAD(P)H) in response to glucose stimulation in islets from control and 2-week diabetic mice (Fig. 4a–c and e). Under control conditions, this signal is dominated by mitochondrial NADH produced by the activity of the TCA cycle^28^. Basal NAD(P)H autofluorescence was substantially elevated in diabetic islets (Fig. 4b and e), as previously shown^29^. However, while elevation of glucose produced a robust increase in NAD(P)H autofluorescence in control islets, this response was much attenuated in 2-week diabetic islets (Fig. 4a and c). In both sets of islets, NAD(P)H autofluorescence was increased by the ATP synthase inhibitor oligomycin and decreased by the mitochondrial uncoupler FCCP. This indicates that the failure of glucose to elevate NAD(P)H in diabetic β-cells is not due to NAD(P)^+^ depletion and that basal mitochondrial metabolism remains at least partially intact in diabetic β-cells.

To determine whether the increase in NAD(P)H autofluorescence represents NADH, we used fluorescence lifetime imaging microscopy (FLIM)^30,31^ (Fig. 4e–g). The mean fluorescence lifetime from enzyme-bound NAD(P)H has been shown to reflect the NADPH-to-NADH ratio^31^. In control islets, the fluorescence lifetime fell in response to 20 mM glucose, indicating that the increased NAD(P)H signal is primarily due to an increase in NADH, rather than NADPH (Fig. 4f). In contrast, no change in fluorescence lifetime was seen in diabetic islets. Further analysis indicated basal levels of both NADH and NADPH are increased in diabetic islets and neither change on glucose elevation (Fig. 4g). This suggests that the ability of glucose to enhance NADH production by the TCA cycle is impaired in diabetic islets.

Failure of NADH to increase in response to acute glucose elevation is predicted to result in a smaller increase in the mitochondrial membrane potential (*ψ*_m_) and to reduce the normal physiological increases in ATP production and oxygen consumption produced by glucose. As previously shown^14^, β-cell mitochondria hyperpolarised in response to 20 mM glucose and were further hyperpolarised by inhibition of the ATP synthase with oligomycin (which prevents dissipation of *ψ*_m_ by aborting H^+^ flux through the ATP synthase) (Fig. 4d). However, the magnitude of the glucose response was significantly reduced in diabetic islets. Collapse of the proton gradient by FCCP produced mitochondrial depolarisation in both cases.

**Fig. 5:**
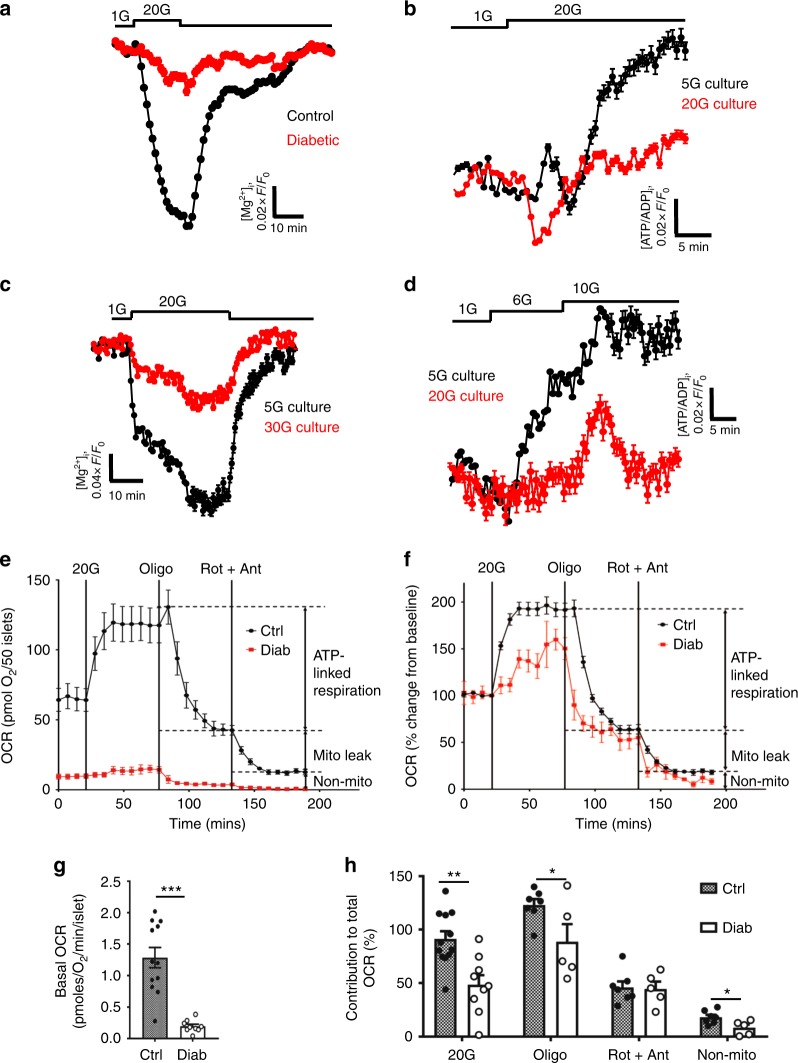
Hyperglycaemia alters islet ATP production and mitochondrial efficiency. **a**, **c** Change in intracellular ATP, as assessed by the reduction in intracellular Mg^2+^ (measured using Mg-green) in response to 20 mM glucose. Data are mean ± s.e.m. **a** Control (black, *n* = 1147 islets) and 2-week diabetic βV59M mouse islets (red, *n* = 423 islets). **c** Control mouse islets cultured at 5 mM (black, *n* = 1228) or 30 mM (red, *n* = 423) glucose for 48 h. **b**, **d** Change in intracellular ATP/ADP ratio (Perceval fluorescence). Data are mean ± s.e.m. **b** In response to 20 mM glucose in control mouse islets cultured for 48 h at 5 mM glucose (black, *n* = 288 islets) or 20 mM glucose (red, *n* = 267 islets). **d**. In response to 6 and 10 mM glucose in non-diabetic human islets cultured for 72 h at 5 mM (black, *n* = 157) or 20 mM (red, *n* = 318) glucose. **e** Oxygen consumption rate (OCR) of control (black) and 2-week diabetic (red) islets at 2 mM glucose and after sequential addition of 20 mM glucose, 5 μM oligomycin and 5 μM rotentone + 5 μM antimycin A. Data points are mean ± s.e.m. (Control, *n* = 7–12 replicates (of 50 islets); Diabetic, *n* = 5–9 replicates (of 50 islets) with 6 animals/genotype). **f** OCR of control (black) and 2-week diabetic (red) islets normalised to percentage of baseline, at 2 mM glucose and after sequential addition of 20 mM glucose, 5 μM oligomycin and 5 μM rotentone + 5 μM antimycin A. Data points are mean ± s.e.m. Same data as in **e** (Control, *n* = 7–12 replicates (of 50 islets); Diabetic, *n* = 5–9 replicates (of 50 islets) with 6 animals/genotype). **g** Basal OCR at 2 mM glucose (basal OCR) of control (hatched) and 2-week diabetic (white) islets. Control, *n* = 12 replicates; diabetic *n* = 9 replicates; 6 animals/genotype. Data points are mean ± s.e.m. ***p < 0.001. Same data as in **e**. **h** Percentage of change in OCR when glucose was raised from 2 to 20 mM (20G), ATP-linked OCR (oligo), OCR required to maintain the mitochondrial leak (rot + ant) and non-mitochondrial OCR (non-mito) in control (hatched) and 2-week diabetic (white) islets. 20 G, 20 mM glucose. Oligo, 5 µM oligomycin. Rot + Ant, 50 µM rotenone + 5 μM antimycin A. Control, *n* = 12 replicates (20G), *n* = 7 (other compounds). Diabetic, *n* = 9 replicates (20 G), *n* = 5 (other compounds). 6 animals/genotype. Same data as in **e**. Data are mean ± s.e.m. *t* test *p < 0.05; **p < 0.01; ***p < 0.001

We have previously shown that, in contrast to control islets, glucose fails to elevate intracellular ATP ([ATP]_i_) in diabetic islets^22^. However, global [ATP]_i_ measurements are confounded because ATP is contained within insulin granules^32^. To confirm that impaired mitochondrial metabolism results in reduced cytosolic ATP levels, we used the fluorescent sensors Mg-green and Perceval to monitor cytosolic ATP/ADP^33,34^. As Mg^2+^ binds with higher affinity to ATP than to ADP, an increase in the ATP/ADP ratio is reflected in a fall in free [Mg^2+^]_i_. Mg-green can be used on freshly isolated islets. In contrast, to enable expression of the genetically encoded sensor Perceval, which measures the cytosolic ATP/ADP ratio directly, islets must be cultured for several days: thus for these experiments we used control islets (mouse and human) cultured at either low or high glucose. With both methods, acute glucose elevation produced a rapid increase in ATP/ADP in control islets but not in 2-week diabetic βV59M islets (Fig. 5a) nor in control mouse islets (Fig. 5b, c) or non-diabetic human islets (Fig. 5d) that had been cultured for 48 h (mouse) or 72 h (human) at high glucose.

These data suggest that mitochondrial metabolism driven by glycolysis is impaired by chronic hyperglycaemia and predict that glucose oxidation by diabetic islets should be reduced. Figure 5e and h shows that this is indeed the case. The oxygen consumption rate (OCR) at basal (2 mM) glucose and the increase stimulated by 20 mM glucose were both substantially lower in diabetic islets than in control islets. Attenuated ATP synthase activity was also observed, as indicated by the reduced respiratory response to metabolic inhibition by oligomycin (Fig. 5h). These metabolic changes are consistent with the smaller glucose-induced rise in NAD(P)H and the ATP/ADP ratio. Subsequent addition of rotenone and antimycin A, which inhibit complex 1 and 3 of the ETC respectively, suppressed OCR to the same degree in both diabetic and control islets, indicating no difference in mitochondrial leak (Fig. 5h). Non-mitochondrial glucose oxidation was lower in diabetic islets (Fig. 5h).

### Effects of diabetes on metabolic pathways

Unlike most cells, β-cells lack the lactate transporter MCT1^35,36^ and have reduced expression of lactate dehydrogenase^36,37^, so that little excess glucose is metabolised to lactate. Thus the rate of glycolysis cannot be determined from measurements of lactate efflux. We therefore measured the rate of glucose utilisation as the release of ^3^H_2_O from [^3^H]glucose^38^. Figure 1b shows that [^3^H]glucose utilisation was significantly reduced in diabetic islets (by ~25%), despite the increase in expression of glycolytic genes and proteins.

**Fig. 6:**
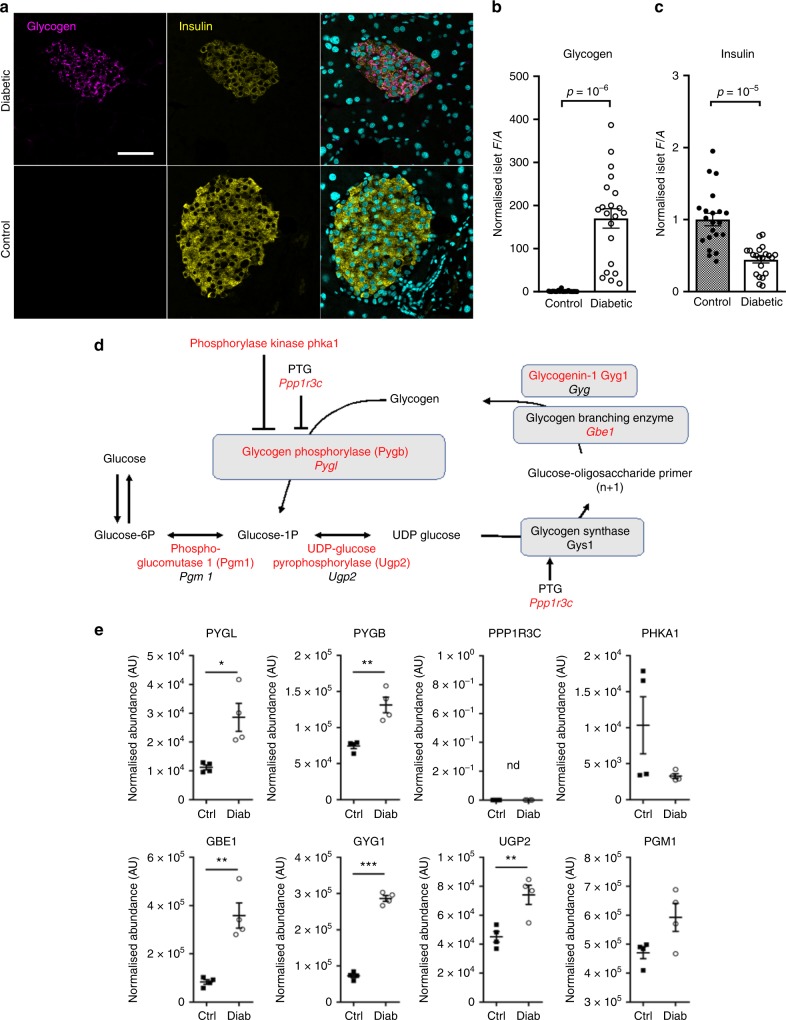
Hyperglycaemia causes profound glycogen accumulation in β-cells. Glycogen and insulin quantification in β-cells of βV59M diabetic animals and controls. Diabetes was induced at 12–13 weeks of age and pancreata were collected 2 weeks after induction, fixed and processed for paraffin embedding and immunohistochemistry. **a** Glycogen (Alexa647, magenta) and insulin (Alexa488, yellow) were detected by immunofluorescence staining in 5-µm sections from diabetic and control mice. Nuclei were stained with SYTOX blue (cyan). Images are representative of three independent experiments. Identical settings were used for confocal imaging of all pictures analysed. Scale bar = 100 µm. For quantification method details (**b**, **c**), see “Methods” section. **b** Glycogen upregulation, quantified as normalised average fluorescence density (*F*/*A*) within the insulin-positive area of the islet. Total number of islets *n* = 21 from *n* = 3 animals (*n* = 7 islets/mouse). Control: 1.0 ± 0.4; Diabetic: 170 ± 23. Error bars show s.e.m., *p* = 10^–6^. (Welch’s *t* test). **c** Insulin was quantified and normalised as for **b**. Control: 1.00 ± 0.09; Diabetic: 0.44 ± 0.04; error bars show s.e.m. *p* = 10^–5^ (Welch’s *t* test). **d** Glycogen pathway. Enzymes or genes indicated in red are increased in diabetic βV59M islets. Enzymes or genes indicated in black are either unchanged in diabetic βV59M islets or were not detected at the protein level. Genes are indicated in italics, proteins in roman type. PTG, protein targeting to glycogen. Phka1, skeletal muscle phosphorylase B kinase alpha subunit. **e** Abundance of the indicated proteins, quantified by mass spectrometry, in islets isolated from control mice (black, Ctrl, *n* = 4) and 2-week diabetic βV59M mice (white, Diab, *n* = 4). Each data point indicates a separate mouse. Mean ± s.e.m. Student’s *t* test (unpaired, two-sided). *p < 0.05, **p < 0.01, ***p < 0.001. nd not detected. PYGL, glycogen phosphorylase (liver type). PYGB, glycogen phosphorylase (brain type). PPP1R3C, protein phosphatase 1 regulatory subunit 3C (also called protein targeting to glycogen or PTG): this protein was not detected but the mRNA increased 2.6-fold (log2fc; *p* = 2.7^e−9^), GBE1, glycogen branching enzyme. GYG1, glycogenin 1. UGP2, UDP-glucose pyrophosphorylase 2. PGM1, phosphoglucomutase-1

Because glucose uptake into β-cells is not rate limiting, yet both glycolytic and mitochondrial glucose metabolism are reduced in diabetic islets, a key question is what happens to the glucose carbons. One possibility is that excess glucose is converted to glycogen^22,39^. Indeed, there was a dramatic increase in glycogen (>150-fold) and a striking upregulation of enzymes involved in glycogen synthesis and its regulation in 2-week diabetic islets (Fig. 6). The marked increase in mRNA (24-fold) and protein (94-fold) levels of the aquaporin channel AQP4 might also reflect glycogen accumulation, which is stored with water.

Pathway analysis indicated marked upregulation of steroid biosynthesis and cholesterol pathways in diabetic islets (Fig. 1, Supplementary Fig. 1). Numerous enzymes involved in cholesterol synthesis were upregulated at the protein level, some very substantially (Supplementary Fig. 2a). These included HMGCoA synthase (22-fold increase), HMGCoA reductase (the principal regulator of cholesterol synthesis, 4.4-fold) and DHCR7 (the final enzyme in cholesterol synthesis, 4.4-fold) (Supplementary Fig. 2a). Expression of many proteins involved in fatty acid (FA) synthesis was also enhanced (Supplementary Fig. 2a). In contrast, genes involved in FA catabolism were downregulated (Supplementary Fig. 2b). Thus some glucose may also be diverted to FA synthesis.

### Metabolic tracing of glucose metabolism in INS-1 cells

To further explore the effect of the observed changes in metabolic gene and protein expression on cellular metabolism, we characterised changes in the abundance and ^13^C-label incorporation of glucose-derived metabolites by gas chromatography (GC)-MS. We used the INS-1 832/13 insulin-secreting β-cell line for these in vitro studies as greater sensitivity can be obtained (due to the larger amount of material available), and they comprise a pure β-cell population (unlike islets). We cultured INS-1 cells at either 5 mM glucose (control) or 25 mM glucose (hyperglycaemia). We refer to these cells as ‘lowG’ cells and ‘highG’ cells, respectively.

**Fig. 7:**
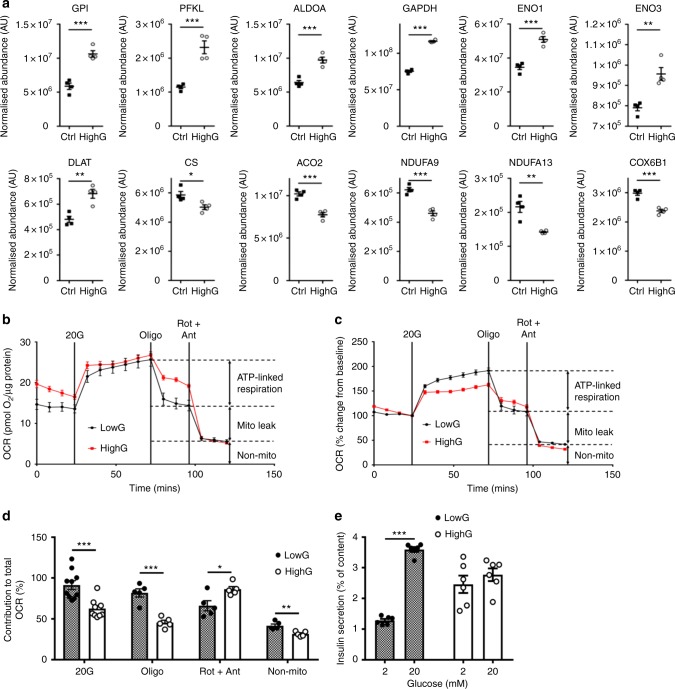
Hyperglycaemia alters oxidative metabolism in INS-1 832/13 cells. **a** Abundance of the indicated proteins, quantified by mass spectrometry, in INS-1 cells cultured at 5 mM (black, Ctrl) or 25 mM (white, highG) glucose. Mean ± s.e.m. (*n* = 4 replicates) and individual data points are indicated. *p < 0.05, **p < 0.01, ***p < 0.001. Student’s *t* test (unpaired, two-sided). Above, enzymes involved in glycolysis. GPI, glucose 6-phosphate isomerase. PFKL, 6-phosphofructokinase, liver type. ALDOA, aldolase A. GAPDH, Glyceraldehyde 3-phosphate dehydrogenase. ENO1, enolase 1. ENO3, enolase 3. Below, mitochondrial enzymes. DLAT, dihydrolipoyllysine-residue acetyltransferase (a component of the pyruvate dehydrogenase complex). CS, citrate synthase. ACO2, aconitase 2. NDUFA9 and NDUF13, subunits 9 and 13 of NADH dehydrogenase:ubiquinone oxidoreductase (Complex 1). COX6B1, Cytochrome c oxidase subunit 6B1. **b**, **c** Oxygen consumption rate (OCR) in INS-1 cells cultured for 48 h at 5 mM (black) or 25 mM (red) glucose, expressed as absolute values (**b**) and as the percentage of change from the OCR baseline in 2 mM glucose (**c**). Data are mean ± s.e.m., *n* = 10 replicates/group (2 and 20 mM Glucose); *n* = 5, other compounds. **d** Percentage of change in OCR when glucose was raised from 2 to 20 mM (20G, *n* = 10 replicates/group), ATP-linked OCR (oligo, *n* = 5 replicates/group), OCR required to maintain the mitochondrial leak (rot + ant, *n* = 5 replicates/group) and non-mitochondrial OCR (non-mito, *n* = 5 replicates/group) in INS-1 832/13 cells cultured for 48 h at 5 mM glucose (hatched, lowG) or 25 mM glucose (white, highG). 20 G, 20 mM glucose. Oligo, 5 µM oligomycine. Rot + Ant, 50 µM rotenone + 5 µM antimycin A. Mean ± s.e.m. Student’s *t* test (unpaired, two-sided). *p < 0.05; **p < 0.01; ***p < 0.001. **e** Insulin secretion, expressed as a percentage of insulin content, for INS-1 cells cultured for 48 h at 5 mM (lowG, hatched) or 25 mM (highG, white) glucose and then exposed to 2 or 20 mM glucose. Mean ± s.e.m. (*n* = 6 experiments). Two-way analysis of variance **p < 0.01

Global protein profiling of INS-1 cells cultured at 5 or 25 mM glucose for 48 h revealed hyperglycaemia-induced changes in protein expression in a similar direction to those found in diabetic islets (upregulation of glycolytic proteins and downregulation of mitochondrial proteins), albeit to a lesser extent (Fig. 7a). Like diabetic islets, glucose-stimulated oxygen consumption was reduced in highG cells (Fig. 7b–d). Chronic hyperglycaemia also reduced GSIS (Fig. 7e).

**Fig. 8:**
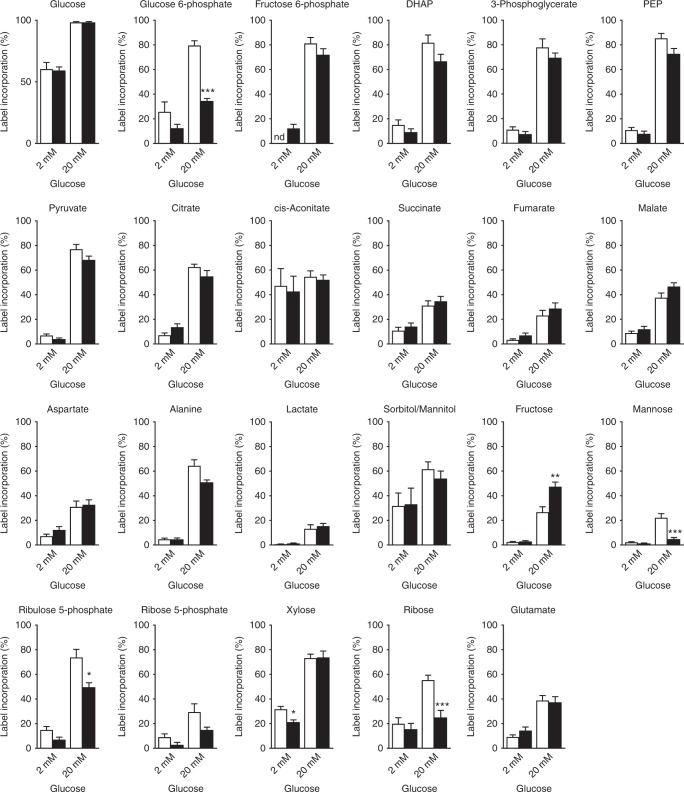
Hyperglycaemia causes changes in [U-^13^C]-glucose labelling. Percentage of label incorporation of the indicated metabolites in INS-1 cells cultured at 5 mM (white) or 25 mM (black) glucose and then challenged with 2 or 20 mM [U-^13^C]-glucose for 30 min. Label incorporation is defined as the proportion of molecules in a metabolite pool containing one or more ^13^C atoms. DHAP, dihydroxyacetone phosphate. PEP, phosphenolpyruvate. Data show mean ± s.e.m. (where from left to right for each metabolite, *n* = 13, 13, 19 and 18). One-way analysis of variance and Bonferroni-corrected Welch’s *t* test. *p < 0.05, **p < 0.01,***p < 0.001, nd not detected

**Fig. 9:**
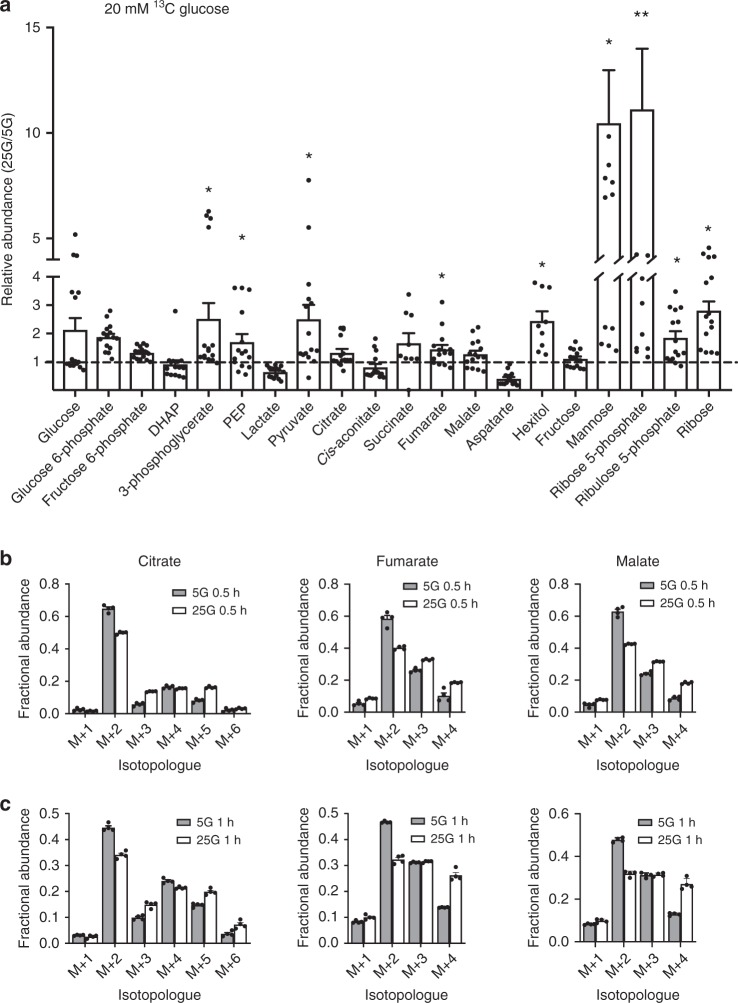
Metabolite abundance and isotopomers in response to hyperglycaemia. **a** Relative abundance of the indicated metabolites in INS-1 cells cultured at 25 mM expressed as a fraction of that in INS-1 cells cultured at 5 mM glucose. All cells were challenged with 20 mM [U-^13^C]-glucose for 30 min. DHAP, dihydroxyacetone phosphate. PEP, phosphenolpyruvate. Hexitol (sorbitol/mannitol). Data are mean ± s.e.m. (*n* = 15 independent samples except for hexitol and succinate where *n* = 9). One-way analysis of variance with Holm–Sidak’s multiple comparisons test, *p < 0.05, **p < 0.01. **b**, **c** Mass isotopomer distributions (MIDs) of the indicated metabolites in INS-1 cells cultured at 5 mM glucose (white bars) or 25 mM glucose (black bars) and then challenged with 20 mM [U-^13^C]-glucose for 30 min (**b**) or 60 min (**c**). Data are derived from a subset of that used in Fig. 8. Mean ± s.e.m.; *n* = 4 independent samples. ‘*M* + *n*’ (where ‘*M*’ is the *m*/*z* of the unlabelled ion) and ‘*n*’ indicates the number of ^13^C atoms in that isotopomer. The data for each isotopomer are expressed as a fraction of the total labelled isotopomers for that metabolite. Since pyruvate and acetyl-CoA contain three and two carbons, respectively, MIDs of +3 are indicative of carbon entry into the TCA through carboxylation of pyruvate, +2, +4 and +6 are indicative of entry through acetyl-CoA (multiple cycles), while +5 indicates that it is derived from both pyruvate carboxylation and acetyl-CoA

For labelling experiments, INS-1 cells were cultured at either 5 mM glucose (lowG cells) or 25 mM glucose (highG cells) for 48 h and then stimulated for 30 min with 2 or 20 mM D-[U-^13^C]-glucose. As expected, elevation of [U-^13^C]-glucose from 2 to 20 mM increased ^13^C incorporation into glucose-derived metabolites (Fig. 8). For most metabolites, the extent of labelling was similar at 20 mM ^13^C-glucose in both lowG and highG cells. Interestingly, label incorporation into glucose 6-phosphate (G6P), ribulose 5-phosphate (Ru5P) and ribose was significantly less in highG cells exposed to 20 mM ^13^C-glucose than in lowG cells. Despite this, the abundance of the PPP metabolites Ru5P and ribose, as well as that of ribose 5-phosphate, were significantly greater in highG cells (Fig. 9a). This suggests that the PPP is upregulated in chronic hyperglycaemia and that the G6P used for this purpose may be derived from a pool of unlabelled glycogen. The increase in ribulose-5-phosphate isomerase and transketolase protein levels (Supplementary Fig. 3) is consistent with the idea that the PPP is upregulated. Like islets, INS-1 cells accumulate glycogen in response to chronic hyperglycaemia^22^. The reduction in mannose labelling at 20 mM ^13^C-glucose in highG cells (Fig. 8), despite its significant increase in abundance (Fig. 9a), may also be indicative of an increased pool of unlabelled G6P. There was no difference in the relative abundance of metabolites between lowG and highG cells at 2 mM ^13^C-glucose (Supplementary Fig. 4).

In contrast, label incorporation into fructose was greater at 20 mM ^13^C-glucose for highG cells than for lowG cells (although the abundance remained the same). This suggests that some glucose carbons are probably channelled into the polyol pathway in chronic hyperglycaemia.

No significant differences were observed in ^13^C-glucose incorporation into TCA cycle metabolites at either 2 or 20 mM glucose in cells cultured under control or hyperglycaemic conditions (Fig. 8). Similarly, with the exception of fumarate, metabolite abundance was not significantly changed in highG cells exposed to 20 mM ^13^C-glucose compared with lowG cells exposed to 20 mM ^13^C-glucose (Fig. 9a). This was despite the significant large increase in pyruvate (with no change in label incorporation) and suggests a block in pyruvate metabolism.

In pancreatic β-cells, ~50% of glucose carbons enter the TCA cycle via pyruvate carboxylase (anaplerosis)^13,27^. We therefore inspected the labelling patterns of individual TCA metabolites in lowG and highG cells after 30 or 60 min exposure to 20 mM [^13^C]-glucose (Fig. 9b). The mass isotopomer distributions of each TCA metabolite indicated the presence of +3 (citrate, malate and fumarate) and +5 (citrate) isotopomers in both lowG and highG cells at both time points. This confirms the presence of pyruvate carboxylation as previously reported^13,27^. Furthermore, the proportion of +3 (citrate, malate, and fumarate) and +5 (citrate) isotopomers was greater in highG cells, which suggests an increase in the relative contribution of pyruvate carboxylation (i.e. anaplerosis) to TCA carbon requirements under hyperglycaemic conditions.

### Other genes and proteins

The effects of diabetes (islets) and chronic hyperglycaemia (INS-1 cells) on the expression of other genes and proteins can be found in the RNA sequencing (RNAseq) and proteomics data uploaded to online databases (see “Data availability”).

## Discussion

Comprehensive transcriptomics and proteomics profiling coupled with analysis of mitochondrial function revealed a rapid and dramatic change in metabolism in diabetic islets (in vivo), with glycolysis being reduced and mitochondrial metabolism very substantially impaired. Similar changes in gene/protein expression and oxidative metabolism were found in INS-1 cells 832/13 exposed to chronic hyperglycaemia (in vitro).

Pathway analysis revealed that glycolysis was among the most significantly upregulated pathways in diabetic islets, with a marked increase in most glycolytic enzymes at both the protein and mRNA levels. Upregulation of selected glycolytic enzymes has also been observed in GK rat islets^21^ and in rat islets and cell lines cultured at high glucose^40,41^. In our study, aldolase B was the most upregulated protein in diabetic βV59M islets, increasing >65-fold. Transcriptomics indicates that aldolase B mRNA is also highly upregulated in rat islets cultured at high glucose^40^ and in β-cells of T2D subjects^42,43^, where it correlates positively with HbA1c and negatively with insulin release^43^. In hepatocytes, aldolase A mainly participates in glycolysis, whereas aldolase B is principally involved in gluconeogenesis^44^. If this is also true in β-cells, it would favour glycogen production. Despite the marked increase in the expression of glycolytic genes and proteins, glucose utilisation in βV59M islets was reduced, by ~25%. This may be a consequence of the observed upregulation of proteins involved in glycogenesis or reflect changes in regulatory control mechanisms (as changes in gene/protein expression do not necessarily reflect a change in activity).

In contrast to most glycolytic enzymes, glucose 6-phosphatase (G6PC2) was downregulated in diabetic βV59M islets. G6PC2 is highly expressed in mouse islets but its functional role is controversial, as it is expected to generate a futile cycle, reducing glycolytic flux, and thereby ATP synthesis and insulin secretion.

Diabetes dramatically impaired glucose-stimulated mitochondrial metabolism in βV59M islets, as evidenced by abrogation of the glucose-stimulated increases in NADH, ATP and oxygen consumption and the marked changes in gene/protein expression. The decreased abundance of many proteins involved in mitochondrial metabolism likely accounts for the impaired metabolism. Diabetes resulted in the coordinated suppression of many TCA cycle enzymes and numerous ETC enzymes in βV59M islets at both the gene and protein levels. The effect on Complex I was especially notable with ~50% of proteins identified being downregulated.

Single-cell transcriptome profiling of human T2D islets has revealed that a number of genes responsible for oxidative phosphorylation and ATP synthesis are downregulated in T2D individuals^19^. Both oxidative phosphorylation and TCA cycle mRNAs and proteins were also downregulated in GK rats at an early stage in the development of diabetes^21^. Our data suggest these changes may be a consequence of the developing hyperglycaemia rather than a result of any underlying mutations/polymorphisms that predispose to diabetes in humans and GK rats.

Our analysis of mitochondrial function indicated that both mitochondrial coupling efficiency and oxygen consumption were impaired in diabetic βV59M islets, leading to a failure of glucose to elevate cytosolic ATP levels and so drive glucose-stimulated insulin release. Mice in which fumarate hydratase deletion causes progressive diabetes show a similar impairment of mitochondrial metabolism and ATP production^23^. Changes in oxidative phosphorylation, PPP and biosynthesis of unsaturated FA were also observed in islets isolated from obese mouse models of diabetes^24^. Our data suggest that hyperglycaemia alone may be sufficient to drive these changes, as FA levels are not elevated in βV59M mice^26^.

The fact that the expression levels of many metabolic proteins, in particular mitochondrial proteins, decreased more than their respective mRNAs in diabetic βV59M islets argues that impaired translation and/or enhanced protein breakdown are more important than reduced transcription. In this context, it may be relevant that the ribosome and proteasome pathways are the second and third most strongly upregulated pathways in the proteomics data (Supplementary Fig. 1).

Our data indicate that in diabetic islets glucose oxidation is very substantially reduced. As glucose freely equilibrates across the β-cell membrane and is not metabolised to lactate^35–37^, the question arises as to the fate of the glucose carbons. The very substantial increase (>150-fold) in glycogen deposition in β-cells suggests that much of the excess glucose ends up as glycogen. The marked increase in the expression of proteins in the pentose phosphate, polyol and FA synthesis pathways suggests that some glucose is also channelled into these pathways. Previous data have also shown that this pathway is operational in clonal β-cells^38,45^. Increased activity of the PPP may contribute to the observed increase in basal NADPH.

A key question is whether the changes we observe in diabetic islets are due to chronic hyperglycaemia or to hypoinsulinaemia, as both are present in βV59M mice (and indeed in all in vivo diabetes models). However, our results clearly indicate that culture of INS-1 832/13 cells for 48 h at 25 mM glucose (which is not associated with hypoinsulinaemia) results in impaired metabolism: in response to subsequent acute glucose elevation, oxygen consumption is reduced and metabolite levels differ. Substantial glycogen accumulation also occurs^22^. Although fewer proteins were identified by proteomics in INS-1 832/13 cells and the changes induced by chronic hyperglycaemia were not as large, their direction was the same as that found for diabetic islets. Changes in selected gene expression also largely matched those observed in diabetic islets^22^. Thus the response of INS-1 832/13 cells to chronic hyperglycaemia resembles that of islets to diabetes. In addition, like diabetic islets, control islets cultured at high glucose failed to elevate ATP in response to glucose stimulation. Consequently, we favour the idea that the metabolic changes we observe are primarily caused by hyperglycaemia.

We observed a few differences between diabetic islets and highG INS-1 832/13 cells. For example, changes in expression of some genes differed: e.g. Cox6a2 was downregulated in diabetic islets (this paper) but upregulated in INS-1 832/13 cells^22^.

Targeted metabolomics of highG INS-1 832/13 cells supports the idea that chronic hyperglycaemia causes metabolic changes in β-cells. Previous metabolomics studies corroborate this view^4^. In highG cells, G6P was labelled substantially less than glucose. This implies that a pool of unlabelled G6P dilutes the ^13^C-G6P; one possibility is that this derives from stored glycogen^46^. In contrast, fructose was labelled more strongly, suggesting that fructose generation via the polyol pathway is increased. The increased hexitol abundance supports this idea.

Our data are in good agreement with earlier transcriptomics data from diabetic human and rodent islets^19,21^. In general, the changes we observed were greater and more extensive than those seen in T2D studies: this may be because T2D patients have better glycaemic control, because their insulin levels are higher or because many are obese and dyslipidaemic (unlike βV59M mice). It may also relate to different sensitivities in the methods employed—in many cases, the changes we observed were greater at the protein than at the mRNA level. Indeed, it is remarkable that so many gene changes are similar, especially given the variation in glycaemia between T2D individuals and the inevitably longer time taken for islet isolation.

Our studies suggest that hyperglycaemia impairs mitochondrial metabolism and reduces the glucose-induced increase in ATP that is required for insulin secretion. This metabolic change has profound functional consequences because mitochondrial metabolism is essential for GSIS^12^. Lack of ATP will impair K_ATP_ channel closure and thereby membrane depolarisation, calcium influx and insulin granule exocytosis. This will further elevate blood glucose and trigger a vicious cycle in which impaired insulin secretion precipitates further glucose elevation. Thus our data provide strong support for the idea that diabetes progression is driven by hyperglycaemia.

**Fig. 10:**
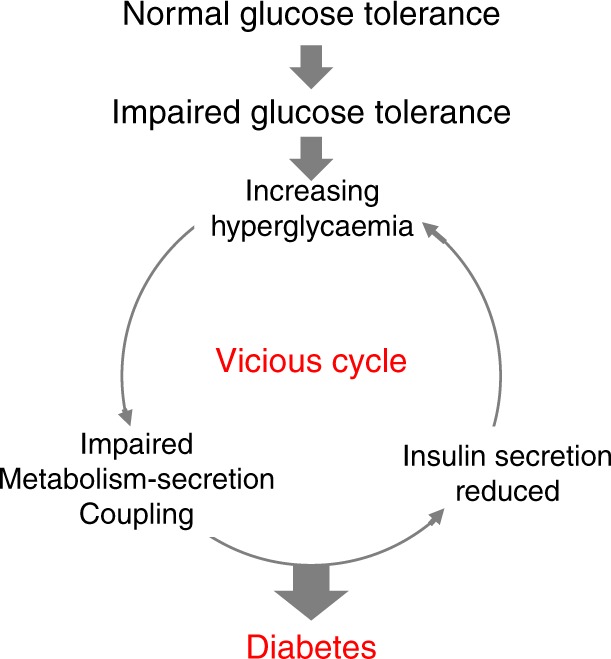
Schematic showing how a small rise in glucose might lead a vicious cycle that progresses to diabetes by progressively impairing β-cell metabolism

We propose that a combination of genetic and lifestyle factors, which may vary between individuals, leads to a small reduction in insulin release and modest elevation of blood glucose (Fig. 10). Chronic hyperglycaemia further impairs insulin secretion, enhancing hyperglycaemia and triggering a vicious cycle that fuels a progressive deterioration in β-cell function and conversion of impaired glucose tolerance to frank diabetes. Because hyperglycaemia is common to all forms of diabetes, this process may be expected to occur in all types of diabetes, including neonatal diabetes and T1D. It is now recognised that β-cells may remain for an extended period after the onset of T1D but fail to release sufficient insulin to control glycaemia^47^: our data suggest that hyperglycaemia may contribute to the impaired β-cell function.

In conclusion, our data provide support for the idea that hyperglycaemia alone is sufficient to produce diabetes progression and demonstrate that the underlying molecular event is a deficit in mitochondrial metabolism. They also emphasise the crucial importance of good glucose control in diabetes in order to maintain β-cell function.

## Methods

### Animal experiments

All animal studies were conducted in accordance with the UK Animals (Scientific Procedures) Act (1986) and approved by the local Department of Physiology Anatomy and Genetics (University of Oxford) ethical review committee. βV59M mice (which hemizygously express the inducible *Kir6.2*-V59M transgene in β-cells) were generated using a Cre-lox approach^22^. Transgene expression was induced by subcutaneous injection of tamoxifen (Sigma, 10 µl/g body weight of 20 mg/ml) in corn oil. As controls, we used tamoxifen-injected wild-type, RIPII-Cre-ER and floxed *Kir6.2*-V59M littermates (pooled). Mice had unrestricted access to water and a regular chow diet (63% carbohydrate, 23% protein, 4% fat; Special Diet Services, RM3). They were maintained on a 12 h light–dark cycle at 21 °C.

Body weight and blood glucose levels were monitored routinely. Blood glucose levels were measured from the tail vein using the Freestyle Lite device and Freestyle Lite test strips (both Abbott). Blood glucose levels were 8.4 ± 0.5 mM in control and 26.7 ± 0.9 mM in diabetic mice. Serum was obtained by incubating whole blood on ice for 30 min, followed by centrifugation at 3000 × *g* and 4 °C for 30 min. The serum was then snap frozen in liquid nitrogen for further analysis.

### Islet isolation

Mice were killed by cervical dislocation. Islets were isolated by injection of 2 ml liberase solution into the bile duct (Liberase TL, Sigma, 0.5 mg/ml in Hanks solution). Pancreas tissue was digested at 37 °C for 16 min. The reaction was stopped by adding 10 ml ice-cold Hanks buffer containing 0.2% bovine serum albumin (BSA; Sigma), followed by 4× pipetting through a 16-G syringe. Islets were hand-picked 4 times and kept in RPMI-1640 medium containing 10% foetal bovine serum (FBS) and 1% Pen/Strep at 37 °C. Freshly isolated islets without culture in RPMI were used for transcriptomics and proteomics analyses.

Human pancreatic islets were isolated from deceased donors under ethical approval obtained from the local human research ethics committee in Oxford. All donors gave informed research consent. Islets were supplied by the Oxford Diabetes Research & Wellness Foundation Human Islet Isolation Facility and isolated according to published protocols^48^.

### INS-1 832/13 cell culture

INS-1 cells were originally developed by Claes Wollheim (Geneva) and supplied by Patrik Rorsman (Oxford). INS-1 832/13 cells were cultured in a humidified atmosphere of 5% CO_2_/95% air at 37 °C in RPMI-1640 medium supplemented with 10% FBS, 1% Pen/Strep, 50 μM β-mercapto-ethanol, 1 mM sodium-pyruvate, 10 mM HEPES and 1% glutamax (standard culture medium; all Sigma). Unless otherwise stated, the glucose concentration was 11 mM. INS-1 832/13 cells were used for metabolomics as ^13^C-glucose labelling is precluded in diabetic animals due to the prevailing high blood glucose (which dilutes the labelled glucose). In addition, greater sensitivity can be obtained due to the greater amount of material available, and INS-1 832/13 constitute a pure β-cell population.

### Insulin secretion

INS-1 832/13 cells were grown to 80% confluency on 24-well plates and cultured in RPMI medium containing 5 or 25 mM glucose for 48 h. They were then washed twice in Tanaka Robertson buffer (118.5 mM NaCl, 2.54 mM CaCl_2_, 1.19 mM KH_2_PO_4_, 4.74 mM KCl, 25 mM NaHCO_3_, 1.19 mM MgSO_4_, 10 mM HEPES) containing 1% BSA and stimulated for 30 min with 2 or 20 mM glucose in the same buffer. The supernatant was removed and cells were harvested in acid ethanol. Insulin levels in the supernatant and cells were determined using an insulin enzyme-linked immunosorbent assay kit (Mercodia) and expressed as percentage of cell content.

### RNA sequencing

RNAseq was performed to identify differentially expressed genes between the control and diabetic mice. Islets were isolated from control mice and 2-week diabetic βV59M mice. Islets from individual mice (3–4 per group) were analysed separately. Total RNA was isolated using Qiazol lysis reagent and the RNeasy Mini Kit (both Qiagen) per the manufacturer’s instructions. RNA concentration was measured using a Qubit 2.0 (Thermo Fisher), and RNA quality was assessed using a bioanalyser (Agilent).

Libraries were prepared and analysed at the Oxford Genomics Centre (Wellcome Trust Centre for Human Genetics, University of Oxford, Oxford, UK): For the library preparation, total RNA quantity and integrity were re-assessed using the Quant-IT RiboGreen RNA Assay Kit (Invitrogen, Carlsbad, CA, USA) and Agilent Tapestation 2200 RNA Screentape. Purification of mRNA, generation of double-stranded cDNA and library construction were performed using TruSeq® Stranded mRNA HT (RS-122–2103) with minor modifications to the manufacturer’s specifications. The following custom primers (25 µM each) were used for the PCR enrichment step: Multiplex PCR primer 1.0 5′-AAT GAT ACG GCG ACC ACC GAG ATC TAC ACT CTT TCC CTA CAC GAC GCT CTT CCG ATC T-3′; Index primer 5′-CAA GCA GAA GAC GGC ATA CGA GAT CAG TGA CTG GAG TTC AGA CGT GTG CTC TTC CGA TCT-3′. Indices were according to the eight base tags developed by WTCHG^49^. Amplified libraries were analysed for size distribution using the Agilent Tapestation 2200 D1000. Libraries were quantified using Picogreen and relative volumes were pooled accordingly. Sequencing was performed as 75-bp paired end read on a HiSeq4000 according to Illumina’s specifications.

### RNAseq data processing and analysis

Raw sequencing reads were aligned to the mouse genome assembly GRCm38 with STAR v2.5.1b^50^ using GENCODE transcriptome annotations (M12, released August 2016). Gene counts were generated using featureCounts^51^ in pair-ended strand-specific mode with multi-mapping, chimeric or multi-overlapping reads excluded.

Differential expression analysis was performed on the gene-level count data using R (version 3.2.2). The analysis was restricted to the 13,362 protein-coding or lincRNA genes, located on the autosomal or X chromosomes, with appreciable expression (counts per million >1) in either all control or all diabetic mice. Differences in the means between the two groups were calculated using the exactTest function in edgeR after normalisation^52^. *p* Values were corrected for multiple testing using the Benjamini–Hochberg procedure.

### Proteomics

Islets were isolated from control or 2-week diabetic βV59M mice, centrifuged briefly at 2000 × *g* and frozen at −80 °C until use. INS-1 cells were cultured at 5 or 25 mM glucose for 48 h, harvested in ice-cold phosphate-buffered saline (PBS), centrifuged briefly at 2000 × *g* and frozen at −80 °C until use. Samples were prepared for LC-MS/MS analysis as described^53^. Briefly, proteins were precipitated with chloroform and methanol after cell lysis in Ripa buffer (Sigma, R0278) and reduction/alkylation with dithiothreitol/iodoacetamide. Proteins were digested with trypsin (Promega) and the resulting peptides were purified on reverse phase material (SOLA SPE, Thermo Fisher). Samples were injected into a nano LC-MS/MS workflow consisting of a Dionex Ultimate 3000 UPLC and an Orbitrap Fusion Lumos (both Thermo Fisher) instrument.

Peptides were separated on an easyspray column (500 mm × 75 µm) with a flow rate of 250 nl/min and a gradient of 2–35% acetonitrile in 5% dimethyl sulfoxide/0.1% formic acid within 60 min. Detailed MS instrument settings are listed in Supplementary Table 2.

LC-MS/MS data was analysed using label-free precursor quantitation in Progenesis QI (Waters, version 3.0.6039.34628) and peptides identified with Mascot v2.7 (Matrixscience) against the UniProt/Swissprot database (2015/11/26). Peptide FDR was adjusted to 1% and additionally all spectra identified with a score <20 were discarded.

### Pathway enrichment analysis

To identify enriched gene sets with shared effects between the transcriptomics and proteomics data, we used the piano package in R (version 3.2.2)^54^. The Hallmark, KEGG and BIOCARTA lists from the MSigDB collections (version 5)^55^ were used as the gene set references. For each data type individually, gene set enrichment was calculated using piano’s consensus scoring approach combining seven different enrichment statistics (mean, median and summed enrichment tests, Fisher’s combined probability test, Wilcoxon rank-sum test, Stouffer’s method and tail strength). From the proteomics and transcriptomics results, the significant and directionally consistent gene sets were identified using only the ‘distinct direction’ results and an FDR < 5% in both data sets.

### Metabolomics

INS-1 cells were plated at 5 × 10^5^ cells/6-well in standard culture medium. The next day, the medium was changed to RPMI-1640 medium containing all supplements and 5 or 25 mM glucose. After 48 h, cells were incubated for 30 mins in Krebs buffer containing 2 mM glucose before being stimulated with Krebs buffer containing 2 or 20 mM d-(U-^13^C]-glucose. The cells in all wells were confluent at the time of measurement. After 30 min, media was quickly removed from each well and plates were transferred to an ice-cold water bath and cells were washed twice with ice-cold PBS. Cells were scraped into ice-cold PBS, transferred to a pre-chilled Eppendorf tube, spun (18,506 × *g*, 4 °C, 90 s) and supernatant removed. Metabolites were extracted by addition of 600 µl chloroform:methanol (2:1, v/v) and subsequent sonication (3 × 8 min, 4 °C, over 1 h) in a water bath sonicator. Samples were spun (18,506 × *g*, 4 °C, 10 min), and the supernatant was transferred to a new tube and dried in a rotary vacuum concentrator. Cell pellets were re-extracted with 600 µl methanol:water (2:1, v/v, containing 1 nmol *scyllo*-inositol (internal standard) and subsequent sonication (8 min, 4 °C). Samples were spun (18,506 × *g*, 4 °C, 10 min), and the supernatant was added to the dried first extract and dried as before. Polar metabolites were separated from apolar metabolites by biphasic partitioning (350 µl chloroform:methanol:water (1:3:3, v/v). Polar metabolites were dried and washed twice with methanol, followed by derivation by methoxymation (20 mg/ml methoxyamine hydrochloride in pyridine (both Sigma Aldrich), room temperature, overnight) and trimethylsilylation (99:1 BSTFA + TMS (Supelco)) for >1 h, before injection onto the GC-MS.

Polar metabolites were analysed by GC-MS (Agilent 7890B-5977A) and identification, abundance and label incorporation of individual metabolites was estimated^56^. In brief, GC-MS was performed using splitless injection (injection temperature 270 °C) onto a 30 m + 10 m × 0.25 mm DB-5MS + DG column (Agilent J&W), with a helium carrier gas, in electron impact ionisation mode. The oven temperature was initially 70 °C (2 min), followed by a temperature increase to 295 °C at 12.5 °C/min and subsequently to 320 °C at 25 °C/min (held for 3 min). GAVIN software^57^ was used for metabolite identification and quantification by comparison to the retention times, mass spectra and responses of known amounts of authentic external standards. Abundance data were corrected for enhanced proliferation at 25 mM glucose by normalising to differences in cell number or protein content.

### Imaging

We used Mg-green (Thermo Fisher Molecular Probes) to monitor cytosolic ([Mg^2+^]_i_). This serves as an indicator of the ATP/ADP ratio, as Mg^2+^ binds with higher affinity to ATP than to ADP. Thus a fall in free [Mg^2+^]_i_ corresponds to an increase in the ATP/ADP ratio. This probe has the advantage that it is pH insensitive and does not require islet culture. We also measured the ATP/ADP ratio using the genetically encoded probe Perceval^33^.

[Mg^2+^]_i_ and NAD(P)H were imaged concurrently using a Zeiss AxioZoom.V16 zoom microscope and 10–14-fold magnification. Mouse islets were preloaded with 6 μM Mg-green in extracellular solution (ECS) supplemented with 6 mM glucose at room temperature for 90 min. ECS contained (in mM) 140 NaCl, 4.6 KCl, 2.6 CaCl_2_, 1.2 MgCl_2_, 1 NaH_2_PO_4_, 5 NaHCO_3_ and 10 HEPES, (pH 7.4, with NaOH). Groups of islets from mice of different genotypes were positioned in an imaging chamber placed on a heated stage (+34 °C) and perifused continuously with ECS (rate 60 μl/min). Mg-green was excited at 500 nm and emission was collected at 535 nm. NAD(P)H was excited at 365 nm and the emission collected at 445 nm. Time-lapse images were collected every 60 s.

Time-lapse imaging of the ATP/ADP ratio in mouse islets was performed using ×103–×143 magnification on a Zeiss AxioZoom.V16 microscope. Islets were infected with an adenovirus (3 × 10^4^ plaque-forming units per islet) delivering Perceval, a recombinant sensor of ATP/ADP^33^. Groups of islets isolated from control and 2-week diabetic βV59M mice were imaged simultaneously 24 h post-infection at glucose concentrations as indicated, with single-cell resolution. Time-lapse images were collected every 30 s, and the bath solution was perifused at 60 µl/min at 34 °C.

Mitochondrial membrane potential (*ψ*_m_) was imaged using TMRE (tetramethylrhodamine ethyl ester; Thermo Fisher, Molecular Probes). Islets were preloaded with 10 nM TMRE for 30 min prior to experiment and 10 nM TMRE was present throughout the experiment. TMRE was excited at 572 nm and emission collected at 630 nm.

Images were analysed using the open-source FIJI software (http://fiji.sc/Fiji). For [Mg^2+^]_i_ and ATP/ADP imaging, timelapse data were analysed individually for each cell. However, NAD(P)H and *ψ*_m_ data were analysed for whole islets, as cell borders could not be easily separated. The numerical time series data were analysed using IgorPro package (Wavemetrics).

Fluorescence lifetime imaging of NAD(P)H was performed on an upright LSM510 microscope (Carl Zeiss) with a 0.3 NA ×10 water-dipping objective using a 650 nm short-pass dichroic mirror and 435–485 nm emission filter. Mouse islets were maintained in Krebs buffer containing 2 or 20 mM glucose. Two-photon excitation was provided by a Chameleon (Coherent) Ti:sapphire laser tuned to 720 nm. Emission events were detected by an external hybrid photomultiplier tube (HPM-100, Becker&Hickl) attached to a time-correlated single-photon counting electronics module (SPC-830, Becker&Hickl). Scanning was performed continuously for 2 min with a pixel dwell time of 1.6 μs. Fluorescence decay parameters were extracted from the data using pixel-by-pixel biexponential fitting with 5 × 5 binning, resulting in 4–5 × 10^3^ photons in each decay curve. NAD(P)H FLIM resolves two exponential components: a short lifetime derived from freely diffusing NADPH and NADH and a longer lifetime derived from NADH and NADPH bound to proteins^29,30^. The lifetime of bound NADH is shorter than that of bound NADPH, and so the mean lifetime reflects the relative balance between their contributions^31^. Differences between mean values of the decay parameters were determined using a two-tailed Student’s *t* test. NADH and NADPH concentrations were estimated using the model proposed by Blacker et al.^31^, combining the fluorescence decay parameters with the mean intensities under each condition, calculated from the integrated photon counts.

### Glucose utilisation

Glucose utilisation was measured as the formation of [^3^H]water from [5-^3^H]glucose in size-matched control and 2-week diabetic islets, using 30 islets/replicate as described by Ashcroft and colleagues^38^. Glucose utilisation rates were calculated as pmol glucose utilised/h/islet.

### Immunohistochemistry

Diabetes was induced in Rip2-CRE/floxedSTOP-Kir6.2-V59M double heterozygous mice at 12–13 weeks of age. Pancreata were collected 2 weeks after induction, fixed in 4% paraformaldehyde and embedded in paraffin. Paraffin sections were rehydrated and subjected to heat-induced epitope retrieval (10 mM citric acid pH6/NaOH, 90–92 °C, 5 min), then permeabilised (PBS 0.5% triton X-100). Prior to staining, sections were incubated in blocking buffer (10% goat serum, 1% BSA, 0.1% Triton X-100 in PBS). Glycogen was detected using mouse anti-glycogen (ref. ^58^, made in house; dilution 1:200), biotin-conjugated goat anti-mouse secondary antibody (BA-9200, Vector Labs, 1:200) and Alexa647-conjugated Streptavidin (S32357, Thermo Fisher Scientific, 1:500). Insulin was detected using guinea pig anti-insulin (A0564, Dako 1:500) and Alexa488-conjugated goat anti-guinea pig secondary antibody (A11073, Thermo Fisher Scientific, 1:500). All staining antibodies and conjugates were diluted in blocking buffer. Nuclear counterstain was performed with SYTOX blue (S11348, Thermo Fisher Scientific, 1:2000). Glycogen and Insulin were quantified by measuring the average fluorescence density (*F*/*A*) within the insulin-positive area of the islet, then subtracting background *F*/*A* in a same-size adjacent section of tissue. Data were normalised to the average *F*/*A* measured for control islets.

### Respirometry

The Seahorse XF24 Extracellular Flux Analyser (Seahorse Bioscience, Copenhagen, Denmark) was used in order to assess a range of metabolic parameters through real-time monitoring of the cellular OCR.

INS-1 832/13 cells were cultured at 5 or 25 mM glucose for 30 h in T-75 cell culture flasks before being seeded at a density of 40,000 cells/well in XF 24-well microplates with standard culture media containing 5 or 25 mM glucose for a further 18 h. Cells were washed in serum-free unbuffered assay medium (Dulbecco’s modified Eagle’s medium (DMEM) 5030, Sigma) containing 2 mM glucose for 1 h prior to measurements being taken. Four baseline measurements were taken at the start of the assay before compounds were injected in order to establish that the basal OCR was stable. Glucose-stimulated respiration was obtained through the addition of 20 mM glucose and mitochondrial efficiency was assessed following injection of compounds that inhibit specific mitochondrial processes: ATP-linked respiration (1 µM oligomycin) and proton leak (0.5 µM antimycin A + 0.5 µM rotenone). Data were normalised to the fourth baseline measurement (100%). The percentage of OCR either stimulated or inhibited following the addition of a compound/substrate was also calculated.

Islets were isolated and incubated overnight in RPMI supplemented with either 11 mM glucose (control islets) or 20 mM glucose (diabetic islets). The glucose concentrations of the culture media were chosen in order to reflect the blood glucose levels recorded in the mice before they were sacrificed. On the day of the assay, islets were seeded at 50 islets/well in XF 24-well islet capture microplates in unbuffered DMEM containing 2 mM glucose and 0.1% FA-free BSA for 1–2 h prior to measurements being taken. As above, 4 baseline OCR measurements were taken before the following substrates/compounds were injected: 20 mM glucose, 5 µM oligomycin, 5 µM rotenone, and 5 µM antimycin A. Data are presented as either pmol O_2_/min/50 islets or were normalised to the fourth baseline measurement (100%). The percentage of OCR either stimulated or inhibited following the addition of a compound/substrate was also calculated.

### Statistics

Unless otherwise stated, data are mean ± s.e.m. of the indicated number (*n*) of mice or replicates. Significance was tested by *t* tests, one-way ANOVA and two-way ANOVA using the Graphpad Prism software, as indicated in the figure legends. Post-test corrections were used as indicated in the legends. Differences between groups were considered statistically significant if *p* < 0.05. RNAseq and proteomics analysis are described above.

### Reporting summary

Further information on research design is available in the Nature Research Reporting Summary linked to this article.

## Supplementary information

Supplementary Information

Reporting Summary

## Data Availability

The raw transcriptomics data sets described in the current study are available in the European Nucleotide Archive (https://www.ebi.ac.uk/ena) under the accession number ERP114395. The mass spectrometric proteomics data have been deposited to the ProteomeXchange Consortium via the PRIDE partner repository with the data set identifier PXD012979 and 10.6019/PXD012979. The authors declare that all data supporting the findings of this study are available within the paper (and its supplementary information files) or can be obtained from the authors upon reasonable request.
