# Supplementary material for: Diabetes causes marked inhibition of mitochondrial metabolism in pancreatic β-cells
Source: Nat Commun. 2019 Jun 6;10:2474. doi: 10.1038/s41467-019-10189-x (PMC6554411; doi:10.1038/s41467-019-10189-x)
Supplement: Supplementary file 1 — Supplementary Information [file 41467_2019_10189_MOESM1_ESM.pdf]

Supplementary Information for

Diabetes causes marked inhibition of mitochondrial metabolism in pancreatic β-cells

Elizabeth Haythorne et al.

Supplementary Fig.1

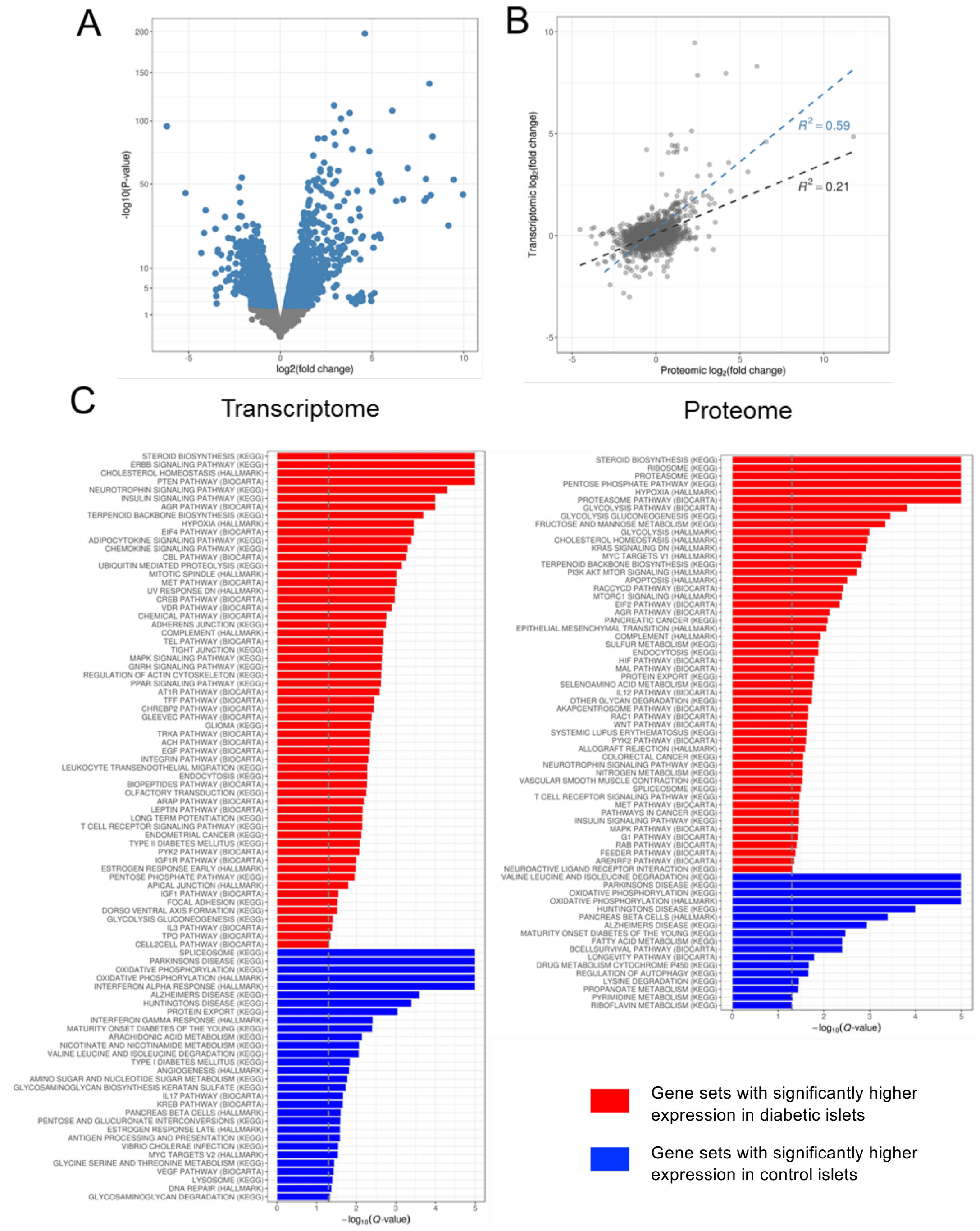

Figure S1. Diabetes causes marked changes in the pancreatic islet transcriptome and proteome

A. Volcano plot of changes in gene expression in βV59M mouse islets after 2 weeks of diabetes. Blue indicates significant changes.
B. Log_2-fold changes between diabetic islets and controls for proteomic (x-axis) and transcriptomic (y-axis) data. The dashed lines show the regression line between the two data sets; r^2, correlation coefficient. The black line indicates all data, the blue line indicates genes and proteins that were significantly changed in both datasets. The fold changes between the significant data have a much stronger correlation.
C. Transcriptomics and proteomics pathway analyses, using Biocarta, KEGG and Hallmark pathways in MSigDB, of data that were directionally consistent and significantly enriched in both the proteomics and transcriptomics data. Red, gene sets with significantly higher enrichment in diabetic islets. Blue, gene sets with significantly higher enrichment in control islets (i.e. downregulated in diabetes).

Supplementary Fig. 2

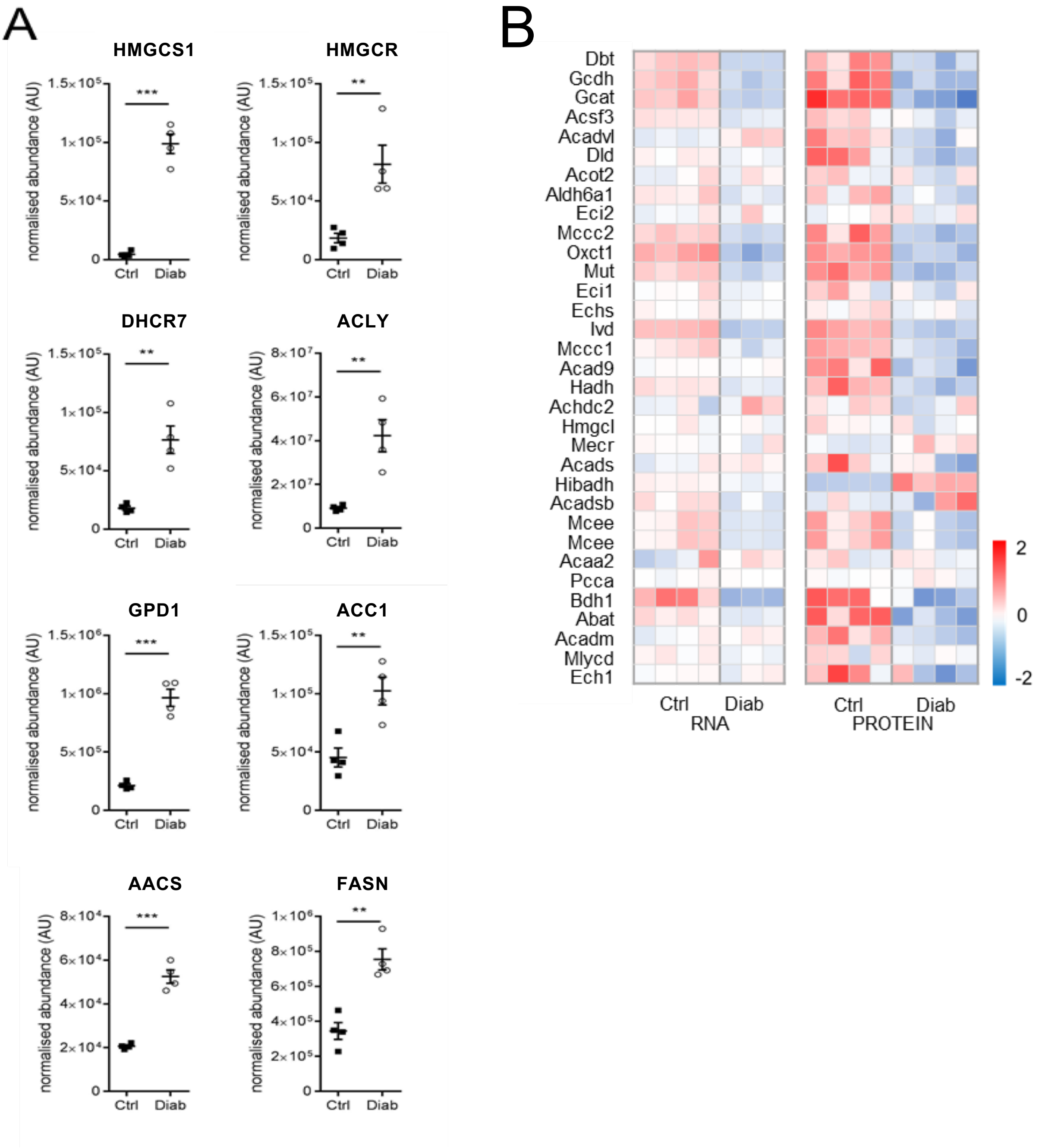

Figure S2. Diabetes increases expression of islet proteins involved in lipid synthesis and decreases expression of mRNA and proteins involved in lipid metabolism

A. Abundance of the indicated proteins involved in lipid synthesis, measured by proteomics, in islets isolated from control (black, Ctrl, n=4) and 2-week diabetic βV59M (white, Diab, n=4) mice. Each data point indicates a separate mouse. Mean±S.E.M. **$p<0.01$, ***$p<0.001$. HMGCS1, 3-Hydroxy-3-methylglutaryl-CoA synthase 1; HMGCR, Hydroxy-3-methylglutaryl-CoA reductase; DHCR7, 7-dehydrocholesterol reductase; ACLY, ATP citrate lyase; GPD1, glycerol phosphate dehydrogenase; ACC1, acetyl-CoA carboxylase 1; AACS, acetoacetyl-CoA synthetase; FASN, Fatty acid synthase.
B. Heat maps of mRNA and protein expression of the indicated lipid metabolism genes in islets isolated from control and 2-week diabetic βV59M mice. Each box corresponds to a different animal. Colour indicates $\log_2$ fold-change.

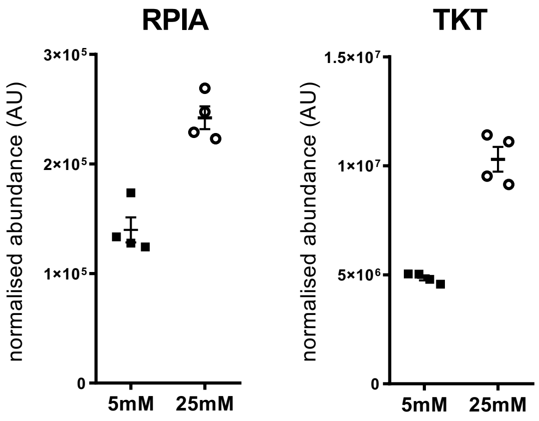

Figure S3. Chronic hyperglycaemia increases expression of pentose phosphate pathway proteins in INS-1 cells

Abundance of the indicated proteins, measured by proteomics, in INS1 cells cultured at 5mM glucose (filled circles, n=4) or 25mM glucose (open circles, n=4). Mean±S.E.M and individual data points for different mice are indicated. Rpia, ribulose-5phosphate isomerase. Tkt, transketolase.

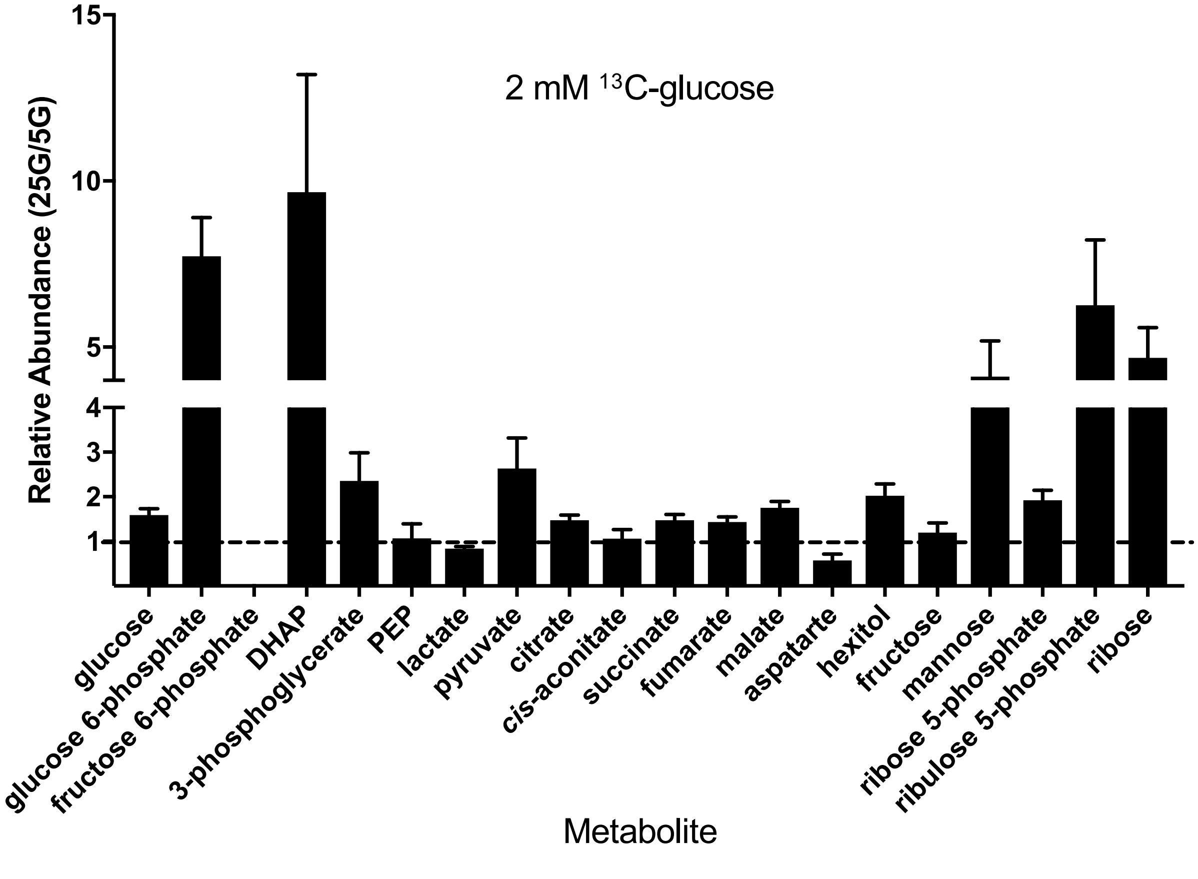

Figure S4. Chronic hyperglycaemia has no significant effect on the levels of metabolites measured in INS-1 cells exposed to 2 mM [U-^{13}C]-glucose for 30min

Relative abundance of the indicated metabolites in INS-1 cells cultured at 5mM expressed as a fraction of that in INS-1 cells cultured at 25mM glucose. All cells were challenged with 2 mM [U-^{13}C]-glucose for 30min. Mean±S.E.M. (n=10 for all samples except hexitol and ribose-5-phosphate where n=6). One-way ANOVA with Bonferroni's multiple comparison. No significant differences were observed.

Supplementary Table 1

Protein/*gene name*	Protein (fold change) diabetic vs control		Gene (Log2 fold change) diabetic vs control	
		p-value		p-value
Aldolase B (*ALDOB*)	65 upregulated	7.80E-08	8.3	6.57E-90
Solute carrier family 5 member 10 (*SLC5A10*)	41 upregulated	1.74E-05	7.94	8.06E-57
Citrate synthase (*CS*)	2.5 downregulated	0.02	0.11	2.87E-01 (ns)
Succinate dehydrogenase (*SDHA*)	2.4 downregulated	0.003	-0.25	2.18E-02
Fumarate hydratase (*FH1*)	3.0 downregulated	0.002	-0.60	5.58E-03
Pyruvate dehydrogenase kinase 1 (*PDK1*)	5.5 upregulated	2.3E-05	2.92	2.08E-93
Pyruvate carboxylase (*PCX*)	1.72 downregulated	0.04	-0.30	2.71E-02
Calcium-binding mitochondrial carrier protein Aralar2 (*SLC25A13)*	1.45 downregulated	0.04	0.52	1.77E-02
Mitochondrial malate dehydrogenase (*MDH2*)	1.9 downregulated	0.0004	-0.35	1.22E-03
Aquaporin channel 4 (*AQP4*)	94 upregulated	7.14E-07	4.61	2.09E-202
HMGCoa synthase (*HMGCS1*)	22 upregulated	2.85E-05	2.18	9.31E-64
HMGCoa reductase (*HMGCR*)	4.4 upregulated	0.002	1.81	2.99E-30
7-Dehydrocholesterol Reductase (*DHCR7*)	4.2 upregulated	0.0002	1.16	3.18E-19
Glycerol-3-phosphate dehydrogenase 1 (*GPD1*)	4.5 upregulated	7.9E-06	1.38	5.9E-22
Glycerol-3-phosphate dehydrogenase 2 (*GPD2*)	1.77 upregulated	0.09 (ns)	1.94	9.29E-18

Supplementary Table 1. Selected metabolic protein and gene changes in diabetic islets. Change in the mean abundance of the indicated protein or gene between control (n=4) and 2-week (n=4) diabetic mice determined by proteomics or transcriptomics respectively. Protein levels are given as fold change, mRNA levels as log2-fold change. ns, not significant.

Supplementary Table 2

Detailed instrument settings for proteomic analysis, using an Orbitrap Fusion Lumos
Orbitrap Fusion Lumos Method Summary

Parameter	Setting
Ion Transfer Tube Temp (°C)	305
Cycle Time (sec)	3
MS1 Detector Type	Orbitrap
Orbitrap Resolution	120K
Scan Range (m/z)	400-1500
Maximum Injection Time (ms)	50
AGC Target	400000
S-Lens RF Level	30
Exclusion duration (s)	60
Filter IntensityThreshold	5000
Isolation Mode	Quadrupole
Isolation Window	1.6
FirstMass	110
ActivationType	CID
Collision Energy (%)	35
MS2 Detector Type	IonTrap
Ion Trap Scan Rate	Rapid
Maximum Injection Time (ms)	300
AGC Target	4000
Inject ions for all available parallelizable time	TRUE
