# Supplementary material for: Diabetes causes marked inhibition of mitochondrial metabolism in pancreatic β-cells
Source: Nat Commun. 2019 Jun 6;10:2474. doi: 10.1038/s41467-019-10189-x (PMC6554411; doi:10.1038/s41467-019-10189-x)
Supplement: Supplementary file 2 — Reporting Summary [file 41467_2019_10189_MOESM2_ESM.pdf]

nature research

Corresponding author(s): Frances Ashcroft

Last updated by author(s): 19 March 2019

Reporting Summary

Nature Research wishes to improve the reproducibility of the work that we publish. This form provides structure for consistency and transparency in reporting. For further information on Nature Research policies, see Authors & Referees and the Editorial Policy Checklist.

Statistics

For all statistical analyses, confirm that the following items are present in the figure legend, table legend, main text, or Methods section.

n/a	Confirmed	
☐	☒	The exact sample size (n) for each experimental group/condition, given as a discrete number and unit of measurement
☐	☒	A statement on whether measurements were taken from distinct samples or whether the same sample was measured repeatedly
☐	☒	The statistical test(s) used AND whether they are one- or two-sided<br>*Only common tests should be described solely by name; describe more complex techniques in the Methods section.*
☒	☐	A description of all covariates tested
☐	☒	A description of any assumptions or corrections, such as tests of normality and adjustment for multiple comparisons
☐	☒	A full description of the statistical parameters including central tendency (e.g. means) or other basic estimates (e.g. regression coefficient) AND variation (e.g. standard deviation) or associated estimates of uncertainty (e.g. confidence intervals)
☐	☒	For null hypothesis testing, the test statistic (e.g. F, t, r) with confidence intervals, effect sizes, degrees of freedom and P value noted<br>*Give P values as exact values whenever suitable.*
☒	☐	For Bayesian analysis, information on the choice of priors and Markov chain Monte Carlo settings
☒	☐	For hierarchical and complex designs, identification of the appropriate level for tests and full reporting of outcomes
☐	☒	Estimates of effect sizes (e.g. Cohen's d, Pearson's r), indicating how they were calculated

Our web collection on statistics for biologists contains articles on many of the points above.

Software and code

Policy information about availability of computer code

Data collection	RNAseq: Illumina HiSeq4000 instrumentation software with bclToFastq v2.17.1.14<br>Proteomics: Orbitrap Fusion Lumos, Tune 2.0.1258.14<br>Metabolomics: Agilent MassHunter Acquisition Software (version B.07.02.1938)<br>Fluorescent imaging: Zeiss Zen 2009, Becker & Hickl SPCM 8.98<br>Immunohistochemistry: Zeiss Zen 2.3 SPI2015 (Black) , Fiji ImageJ 1.51s
Data analysis	Transcript analysis: STAR v2.5.1b, Picard Tools v2.1.1, Subread v1.5.0-p2,<br>Differential expression analysis: biomaRt v2.26.1, edgeR v3.12.1, limma v3.26.9, R v3.2.2<br>Pathway analysis: piano v1.10.2, R v3.2.2<br>Proteomics: Progenesis QI (Waters, version 3.0.6039.34628), Mascot v2.7<br>Metabolomics: GAVIN<br>Fluorescent imaging: Fiji ImageJ 1.52a, IgorPro, Becker & Hickl SPCImage 3.0.8<br>Immunohistochemistry: Fiji ImageJ 1.51s<br>Statistics: GraphPad Prism 6.0

For manuscripts utilizing custom algorithms or software that are central to the research but not yet described in published literature, software must be made available to editors/reviewers. We strongly encourage code deposition in a community repository (e.g. GitHub). See the Nature Research guidelines for submitting code & software for further information.

Data

Policy information about availability of data

All manuscripts must include a data availability statement. This statement should provide the following information, where applicable:

- Accession codes, unique identifiers, or web links for publicly available datasets
- A list of figures that have associated raw data
- A description of any restrictions on data availability

The raw transcriptomic datasets described in the current study are available in the at the European Nucleotide Archive (https://www.ebi.ac.uk/ena) under the accession number ERP114395
The mass spectrometry proteomics data have been deposited to the ProteomeXchange Consortium via the PRIDE partner repository with the dataset identifier PXD012979 and 10.6019/PXD012979

Field-specific reporting

Please select the one below that is the best fit for your research. If you are not sure, read the appropriate sections before making your selection.

☒ Life sciences ☐ Behavioural & social sciences ☐ Ecological, evolutionary & environmental sciences

For a reference copy of the document with all sections, see nature.com/documents/nr-reporting-summary-flat.pdf

Life sciences study design

All studies must disclose on these points even when the disclosure is negative.

Sample size	The samples sizes for each experiment are stated in the Methods section and the Figure legends of the paper
Data exclusions	No data were excluded.
Replication	Similar transcriptome data have been reported by other authors for the effects of chronic hyperglycemia on pancreatic islets and clonal beta-cells.
Randomization	Randomization was not possible as samples were allocated into experimental groups based on genotype or cell culture conditions (high, low glucose)
Blinding	The investigator was blinded to the genotype of the mouse, but not otherwise.

Reporting for specific materials, systems and methods

We require information from authors about some types of materials, experimental systems and methods used in many studies. Here, indicate whether each material, system or method listed is relevant to your study. If you are not sure if a list item applies to your research, read the appropriate section before selecting a response.

Materials & experimental systems

n/a	Involved in the study	
☐	☒	Antibodies
☐	☒	Eukaryotic cell lines
☒	☐	Palaeontology
☐	☒	Animals and other organisms
☒	☐	Human research participants
☒	☐	Clinical data

Methods

n/a	Involved in the study	
☒	☐	ChIP-seq
☒	☐	Flow cytometry
☒	☐	MRI-based neuroimaging

Antibodies

Antibodies used	All antibodies used, and the suppliers, are described in the Materials and Methods section under Immunohistochemistry
Validation	Commercial antibodies were validated by the manufacturer. The glycogen antibody was validated in reference 57

Eukaryotic cell lines

Policy information about cell lines

Cell line source(s)	INS-1 832/13 cells were originally developed by Prof Claes Wollheim (Geneva) and were supplied by Patrik Rorsman (Oxford)

Authentication	INS-1 832/13 cells were authenticated functionally by examining glucose-stimulated insulin secretion, and genetically by examining expression of beta-cells genes such as Pdx1, Nkx6.1, MafA, MafB and Pax6.
Mycoplasma contamination	Cell lines were tested for mycoplasma when they first arrived in the laboratory.
Commonly misidentified lines (See ICLAC register)	*Name any commonly misidentified cell lines used in the study and provide a rationale for their use.*

Animals and other organisms

Policy information about studies involving animals; ARRIVE guidelines recommended for reporting animal research

Laboratory animals	Male and female mice at 12 weeks of age, of the C57BL6/J background strain, which are wild-type or carry the Kir6.2-V59M and RIP2-CreER transgenes as indicated in the Methods.
Wild animals	N/A
Field-collected samples	N/A
Ethics oversight	All animal procedures were conducted in accordance with the UK Animals (Scientific Procedures) Act (1986) and were approved by the Department of Physiology, Anatomy and Genetics local ethical review committee.

Note that full information on the approval of the study protocol must also be provided in the manuscript.
